# A deep-learning technique for phase identification in multiphase inorganic compounds using synthetic XRD powder patterns

**DOI:** 10.1038/s41467-019-13749-3

**Published:** 2020-01-03

**Authors:** Jin-Woong Lee, Woon Bae Park, Jin Hee Lee, Satendra Pal Singh, Kee-Sun Sohn

**Affiliations:** Faculty of Nanotechnology and Advanced Materials Engineering, Sejong University, Seoul, 143-747 Republic of Korea

**Keywords:** X-ray diffraction, Inorganic chemistry, Computational methods

## Abstract

Here we report a facile, prompt protocol based on deep-learning techniques to sort out intricate phase identification and quantification problems in complex multiphase inorganic compounds. We simulate plausible powder X-ray powder diffraction (XRD) patterns for 170 inorganic compounds in the Sr-Li-Al-O quaternary compositional pool, wherein promising LED phosphors have been recently discovered. Finally, 1,785,405 synthetic XRD patterns are prepared by combinatorically mixing the simulated powder XRD patterns of 170 inorganic compounds. Convolutional neural network (CNN) models are built and eventually trained using this large prepared dataset. The fully trained CNN model promptly and accurately identifies the constituent phases in complex multiphase inorganic compounds. Although the CNN is trained using the simulated XRD data, a test with real experimental XRD data returns an accuracy of nearly 100% for phase identification and 86% for three-step-phase-fraction quantification.

## Abstract (web summary)

Identifying the composition of multiphase inorganic compounds from XRD patterns is challenging. Here the authors use a convolutional neural network to identify phases in unknown multiphase mixed inorganic powder samples with an accuracy of nearly 90%.

## Introduction

We have recently discovered many novel inorganic functional materials by employing powder X-ray powder diffraction (XRD) analysis. These materials include phosphors for solid-state lighting^1–3^ and cathodes for rechargeable batteries^4–6^. One of the most frequently faced situations in the process of materials discovery based on the powder XRD technique involves the identification and quantification of unknown multiphase compounds. It would be arduous, however, for even a well-trained expert with the advantage of well-established computational tools to complete both the constituent phase identification and the ensuing phase-fraction estimation for a sample consisting of a grungy, multiphase mixture. Despite the existence of a promising level of expertise, the prompt identification of constituent phases from an intricate multiphase mixture would be complicated when using conventional rule-based data analysis tools such as commercially available computational software packages^7–9^. Here we propose providing lay persons who are not experts with a facile, prompt protocol for the quantitative identification of constituent phases in unknown multiphase mixtures.

Deep-learning technologies have achieved a respected position in the materials research community^10–19^ and could make it possible to accomplish a dream protocol that would enable instantaneous phase identification of samples of unknown mixtures. Within the framework of theoretical crystallography-based powder XRD analysis, the XRD pattern is interpreted as discrete intensity data vs. 2*θ* in reciprocal space and thereafter backward-Fourier-transformed into the electron density in real space, and thereby structural information of interest can be extracted from the XRD pattern. The backbone algorithm used in all the commercially available software relies on this crystallography principle. In parallel with such a theoretical basis, some practical logics to separate each of the independent peaks are incorporated in the algorithm by employing adjustable parameters such as peak functions, background, etc. In contrast to such a rule-based traditional approach, the deep-learning approach adopts a completely different principle. The XRD pattern is considered as nothing more than a one-dimensional image in the deep-learning approach and a deep convolutional neural network (CNN) is employed and trained to learn underlying features from a large number of XRD patterns. The underlying features can scarcely be understood by any logics that are used for a traditional approach. Eventually, the deep-learning approach gives rise to swiftness, simplification, and ease of use.

We have very recently developed a symmetry-classification CNN model that can be used to extract a crystal system, extinction group, and space group from a powder XRD pattern^20^. Simulated XRD patterns for 150,000 entries registered in the inorganic compound structure database (ICSD) were used to train the CNN model. This previous report is appreciated as one of the early-stage deep-learning approaches in the crystallography research field as evidenced by the ensuing reviews on deep learning for crystal structure prediction^11^. Rather than the previous symmetry classification for a single-phase compound, herein we introduce a more pragmatic CNN model to sort out phase identification (so-called phase search/match) for unknown multiphase mixture powder samples consisting of several phases, which are more frequently confronted by materials scientists and engineers during ordinary materials research activities.

There have been a number of reports dealing with machine learning (ML) attempts in XRD-based materials research^20–34^ and ML is known to be very powerful when associated with high-throughput experiments^30–34^. In particular, these include a prestigious ML algorithm for XRD-based phase matching based on a convolutive non-negative matrix factorization, the so-called AgileFD^35,36^. What discriminates our deep-learning approach from the general ML approaches lies in the fact that our deep-learning approach aims to preclude any type of human expert intervention and finally to outperform traditional rule-based approaches. Here we are dealing with large datasets (800,942 or 183,521 XRD patterns per a dataset), deep architecture, and the prevention of handcrafted data reduction, whereas most of the ML approaches (even alleged as deep learning) remain restricted within a somewhat vicious circle consisting of small-sized training datasets, shallow artificial neural network architectures, and the excessive feature engineering that is based on human knowledge, all of which impart ML-based analyses with no merit by comparison with rule-based analysis.

A large number of unidentified mixture samples would appear when a combinatorial synthesis process was employed in a quaternary composition of Sr, Al, Li, and O. The philosophy behind the choice of the quaternary composition is based on the fact that some promising luminescent materials for use in light-emitting diode (LED) applications have been recently discovered in a similar composition pool^37–39^. Enormous efforts are required for instantaneous phase identification, while screening a large number of unidentified mixture samples in the quaternary composition pool. The deep-learning-based approach would tremendously facilitate the entire process, and make it comparable to either the conventional rule (logic)-based phase identification protocol or the conventional ML-based approach.

In this regard, we propose a deep CNN model that enables prompt and accurate phase identification of mixtures of inorganic samples, which is compatible with any of the existing rule-based computational tools. We prepare a total of three large datasets including simulated 1,785,405 powder XRD patterns using 170 ICSD entries restricted to the quaternary composition pool. Using these XRD datasets, we train the deep CNN model to act as a promising phase identification platform. Although the CNN is trained using the simulated XRD data, a test with real experimental XRD data returns an accuracy of nearly perfect for phase identification.

## Results

### Phase identification for Dataset_800k_org

**Fig. 1:**
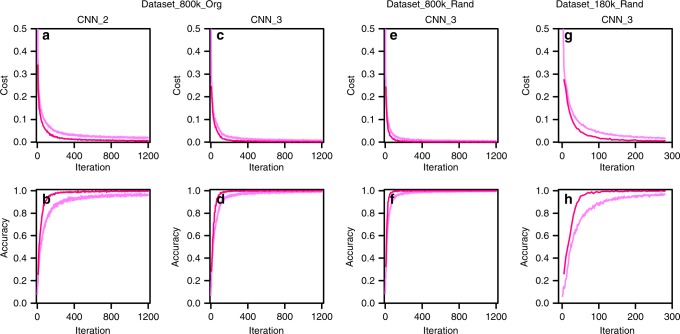
The phase identification result. The training loss/accuracy (dim pink lines) and the validation loss/accuracy (deep pink lines) for both the CNN_2 (**a**, **b**) and CNN_3 (**c**, **d**) trained with Dataset_800k_org, CNN_3 (**e**, **f**) with Dataset_800k_rand, and CNN_3 (**g**, **h**) with Dataset_180k_rand are plotted as a function of the iteration number.

The training loss and accuracy, and the validation loss and accuracy are plotted as a function of the iteration number for both CNN_2 and CNN_3 architectures trained with Dataset_800k_org in Fig. 1a–d. The validation accuracy reached nearly 100% for both the CNNs. The validation loss was decreased to 0.007 and 0.0018 for CNN_2 and CNN_3, respectively. The training loss and accuracy was slightly exacerbated by comparison with the validation loss and accuracy due to a dropout rate of 50%. After completion of the training process, we tested the trained CNN models using a hold-out test dataset that did not have overlap the training dataset. We executed one test dataset that consisted of 100,000 simulated XRD patterns and a second using 100 real experimental XRD patterns measured in the lab. In the former case, the test accuracy reached nearly a perfect level, i.e., 99.60% and 100% for both CNN_2 and CNN_3, respectively. The actual 50-mixture samples were produced by blending three compounds (Li_2_O-SrO-Al_2_O_3_) with various relative fractions and another set of 50-mixture samples was produced by blending another set of three compounds (SrAl_2_O_4_-SrO-Al_2_O_3_). Thereafter, these real XRD patterns were used for the test. The test results for the Li_2_O-SrO-Al_2_O_3_ dataset also showed a perfect match with a test accuracy of 100% for both CNN_2 and CNN_3. On the other hand, the test accuracy deteriorated slightly but still promising in the case of the SrAl_2_O_4_-SrO-Al_2_O_3_ dataset.

Table 1 shows the test accuracy for the simulated and the real XRD test datasets for CNN_2 and CNN_3 architectures. The test accuracy was considered nearly perfect when the simulated test dataset was employed both for the CNN_2 and CNN_3 architectures. Besides the hyper-parameter settings used for the CNN_2 and CNN_3 architectures, some others also showed a comparable test results, for instance, when the kernel (filter) size was doubled (or halved) the same accuracy (nearly 100%) was obtained. As mentioned in the Methods section, the pooling (and stride) scheme was varied from an unconventional to a conventional setting, and both settings returned the same accuracy of nearly 100%. Unfortunately, there is no rigorous principle for determining the hyper-parameters with a proper architecture, although Bayesian optimization has been recently used for this sake^40–43^. The architectures given here were chosen on a trial-and-error basis. The proposed architectures and hyper-parameters were determined after trying as many plausible versions as possible.

**Table 1:** Phase identification test result.

Dataset	Dataset_800k_Org		Dataset_800k_Rand	Dataset_180k_Rand
CNN architecture	CNN_2	CNN_3	CNN_3	CNN_3
Simulated XRD test dataset				(23,000 patterns)
100,000 Patterns	99.60%	100%	100%	99.76%
Real XRD test dataset				
Li_2_O_SrO_Al_2_O_3_ (50 patterns)	100%	100%	100%	98.67%
SrAl_2_O_4__SrO_Al_2_O_3_ (50 patterns)	97.33%	98.67%	98%	97.33%

The phase identification test results from both the simulated (hold-out) and real test datasets for CNN_2 and CNN_3 trained with Dataset_800k_org, Dataset_800k_rand, and Dataset_180k_rand. The CNNs were trained for two epochs

It is evident that the test accuracy never worsened when the actual XRD data were used. The real test dataset for Li_2_O-SrO-Al_2_O_3_ ternary mixtures led to a perfect level for test accuracy (100% for both CNN_2 and CNN_3). On the other hand, the test accuracy of the real test dataset for the SrAl_2_O_4_-SrO-Al_2_O_3_ ternary mixtures was slightly decreased to 97.33% for CNN_2 and 98.67% for CNN_3. When scrutinizing the test results for the SrAl_2_O_4_-SrO-Al_2_O_3_ dataset, however, an interesting finding was detected. Every misprediction included only a single mismatched phase, Sr_4_Al_14_O_25_, for both the CNN_2 and CNN_3 tests, whereas the other two were correctly matched with the ground truth. Although we used a commercially available SrAl_2_O_4_ (SAO) powder sample purchased from Nemoto, Co. Ltd., it was not a single-phase compound but involved a certain amount of an impurity phase that was determined to be Sr_4_Al_14_O_25_. This implies that the misprediction and the slightly deteriorated test accuracy did not originate from any errors in our CNN models, but rather clearly indicates that the CNN model worked perfectly, which suggests a test accuracy of 100%.

To detect the impurity in the commercially available SAO sample, we carefully analyzed the real XRD pattern for the SAO using conventional analysis tools such as the X’pert^8^ and FullProf^9^ software programs. Rietveld refinement precisely revealed that the commercially available SAO sample certainly involved the Sr_4_Al_14_O_25_ impurity at a weight % of 15, as shown in Supplementary Fig. 1. It should be noted that it took several hours for an expert with 10 years of experience to finish these accurate procedures for the phase matching and the ensuing Rietveld refinement. However, our CNN models completed the same task in less than a second.

### Phase identification for Dataset_800k_rand/Dataset_180k_rand

The Dataset_800k_org might raise concerns that portions of the test data were nearly identical to data in the training dataset so that there could be information leakage concerns, since fixed (evenly distributed) ternary and binary compositions were adopted in the blending process. To rule out the concerns of information leakage we separately trained CNN_3 by employing Dataset_800k_rand and Dataset_180k_rand, which were prepared based on a completely random choice of compositions. As a result, the equivalent loss and accuracy was also attained as was the previous case that Dataset_800k_org was used. This result was included in Fig. 1e–h and in Table 1. Both the simulated test dataset and the real Li_2_O-SrO-Al_2_O_3_ test dataset led to nearly 100% accuracy when trained with Dataset_800k_rand. When trained with Dataset_180k_rand, the simulated test dataset and the real test dataset gave rise to a slight degradation in the test accuracy. The fact that the phase identification performance based on these two auxiliary datasets (Dataset_800k_rand and Dataset_180k_rand) was equivalent to that for the original dataset (Dataset_800k_org) also backed up the validity of the original dataset (Dataset_800k_org), which rescinded the concerns about information leakage.

When considering the issue of the similarity between the training and test datasets, the type of synthetic training dataset used would never matter as far as the CNN model worked with real data testing, because we are aware of the conspicuous distinction between the synthetic training dataset and the experimentally measured real dataset. It is evident that the real test dataset differs significantly from the simulated training dataset, but such a distinct real test dataset continued to show nearly 100% accuracy for phase identification. Despite the excellent accuracy of the real data, the inordinate performance of our CNN approach could be partly due to a certain degree of information leakage caused by the similarity between the training and test datasets.

### Phase fraction predictions for Dataset_800k_org

Even when considering the conventional rule-based XRD analysis, it would be difficult to correctly evaluate the relative fraction of each constituent phase in a mixture sample compared with use of the relatively easier process for only phase identification. This is also true for the data-driven XRD analyses. The simple phase identification resulted in nearly perfect performances for both the simulated and real dataset tests. As shown in Table 2, however, the test accuracy was slightly decreased in the constituent-phase-fraction prediction. In fact, the exact estimation of phase fraction is impossible, and only a rough measure is possible. Even the simplified three-level-phase-fraction prediction has never reached 100% accuracy.

**Table 2:** Three-step-phase-fraction prediction test result.

CNN Architecture	CNN_2F	CNN_3F	CNN_4F	CNN_5F	CNN_6F
Simulated XRD test dataset (Dataset_800k_Org)					
100,000 Patterns	94.39%	97.19%	97.64%	98.08%	98.13%
Real XRD test dataset					
Li_2_O_SrO_Al_2_O_3_ (50 patterns)	76.00%	80.00%	83.33%	86.00%	82.67%

The three-level-phase-fraction prediction test results based on Dataset_800k_org. The CNNs were trained for 10 epochs

When we designed the training dataset (Dataset_800k_org), we inevitably had to assign a large number of thoroughly different ternary (or binary) mixtures to reach an identical relative fraction, because fixed (evenly distributed) ternary (or binary) compositions were adopted for each of the 8436 (or 703) ternary (or binary) systems made of 38 classes. More importantly, we had only 21 (or 9) XRD patterns to be used for the constituent-phase-fraction prediction for a particular ternary (or binary) system. A similar situation was also expected for the other training datasets (Dataset_800k_rand and Dataset_180k_rand). This implies that the dataset size was too small to achieve a satisfactory phase-fraction regression. Consequently, a simple regression model would never work when employing the present training dataset, which meant the test result was far from complete. The regression loss (mean square error) for the test was at best 0.01 when trained with Dataset_800k_org.

We transformed the regression problem into a classification problem so as to achieve a rough, but acceptable prediction of the fraction of constituent phases residing in an unknown mixture sample. We compartmentalized the relative phase-fraction values into three levels: low (0 ~ 0.33), intermediate (0.33 ~ 0.66), and high (0.66 ~ 1). As a result, the phase-fraction prediction model turned into a classification model, which amounted to a so-called three-level-fraction prediction model, and the number of nodes in the output layer increased to 114. The CNN_2 and CNN_3 architectures were adopted, but with the output layers altered, which meant that we re-designated the slightly altered CNNs for a three-level-fraction prediction that we refer to here as CNN_2F and CNN_3F. In addition, we introduced three more CNNs with deeper architectures, e.g., four-, five-, and six-convolutional layers denoted as CNN_4F, CNN_5F, and CNN_6F, respectively. To improve the predictability of the phase fraction, finer divisions could be available such that four- and five-level divisions, and so forth, could be accepted, which would lead to 152 and 190 nodes in the output layer.

**Fig. 2:**
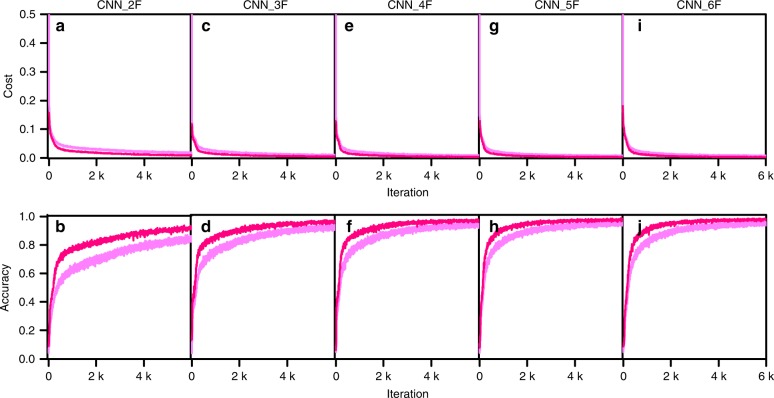
The three-level-phase-fraction prediction result trained with Dataset_800k_org. The training loss/accuracy (dim pink lines) and the validation loss/accuracy (deep pink lines) plotted as a function of the iteration number up to the 10th epoch for the CNN_2F (**a**, **b**), CNN_3F (**c**, **d**), CNN_4F (**e**, **f**), CNN_5F (**g**, **h**), and CNN_6F (**i**, **j**) architectures.

The training loss/accuracy and the validation loss/accuracy for the three-level-fraction prediction, based on the CNN_2F, 3F, 4F, 5F, and 6F architectures trained with Dataset_800k_org are plotted as a function of the iteration number in Fig. 2. The validation accuracy increased to >97%, whereas the validation loss decreased to 0.004 for all three-level-fraction prediction CNNs except for the CNN_2F. The CNN_6F led to the highest validation accuracy of 98.13% at the 10th epoch and showed the most facilitated learning by reaching 97% at the 5th epoch, which is earlier than any others. Only two epochs (1200 iterations with a batch size of 1000) were sufficient to reach a much higher level (100%) of validation accuracy for the simple phase identification. However, 10 epochs (6000 iterations with a batch size of 1000) were required to reach 98.08% for the three-level-prediction of a phase fraction.

The fully trained CNN models for the three-level-phase-fraction prediction were tested using the hold-out test datasets, i.e., the test dataset consisting of 100,000 simulated XRD patterns and the 50 real XRD patterns only for Li_2_O-SrO-Al_2_O_3_. We precluded the other test dataset for SrAl_2_O_4_-SrO-Al_2_O_3_ due to the impurity complications mentioned above. Table 2 shows the summarized results from the test. In the case of the simulated XRD patterns, the test accuracy reached 98.13% for the CNN_6F architecture. The real Li_2_O-SrO-Al_2_O_3_ test dataset led to a lower level of accuracy compared with those for the simulated test dataset for all CNN architectures. These test accuracies for the phase-fraction prediction are acceptable from a practical point of view.

The three-level-prediction can be used to estimate a rough measure of the exact phase fraction. As more finely divided levels would lead to a more accurate fraction prediction, we trained three additional CNNs, which incorporated four- and five-level fraction predictions using basic CNN_2F, CNN_3F, and CNN_6F architectures. The five levels (0–20%, 20–40%, 40–60%, 60–80%, and 80–100%) would allow for more plausible phase-fraction prediction than the three-level phase-fraction prediction. The training was retarded by comparison with the three-level prediction and, therefore, we stopped the training at the 10th epoch. If the fraction level was more finely divided, then the number of nodes at the output layer in the CNN would increase and in turn the number of weights would also increase. Therefore, more epochs would be required to avoid deterioration of the test accuracy. The training loss/accuracy and the validation loss/accuracy for the four- and five-level fraction predictions based on the CNN_2F, CNN_3F, and CNN_6F architectures are plotted to the 10th epoch in Supplementary Fig. 2a-j and the test accuracies are given in Supplementary Table 1. The test accuracy for the five-level phase-fraction prediction based on the CNN_2F, 3F, and 6F architectures were 77.65%, 92.44%, and 94.76%, respectively.

To construct deeper CNN architectures for the five-level phase-fraction prediction, the pursuit of alternative concepts in architecture could be a better option to achieve better performance. The inception net^44,45^ deserved to be adopted in this regard. The performance of the inception net is known to be more promising in comparison with the conventional multi-layered CNNs^44,45^. The architecture of the inception net adopted in the present investigation consists of the existing convolutional layers and additional layers called an inception module that approximates a sparse CNN with a normal dense construction. CNNs with three and six inception modules (referred to as CNN_3I and CNN_6I) were adopted for five-level phase-fraction prediction and the CNN_6I led to the highest test accuracy of 95.90%. The inception net used for the five-level prediction is schematically represented in Supplementary Fig. 3. The training, validation, and test results for CNN_3I and CNN_6I are summarized in Supplementary Fig. 2k~n and Supplementary Table 1. It should be noted, however, that the real test data accuracy deteriorated significantly in the five-level phase-fraction prediction, which was much worse than results from the three-level phase-fraction prediction. Further efforts to create more realistic synthetic XRD patterns are required to improve the real data testing for the five-level phase-fraction prediction in future work.

### Real data test

Simple phase identification resulted in equal and nearly perfect performances for both the simulated and real dataset tests. As shown in Table 2, however, the test accuracy was slightly decreased when the real XRD patterns were used for the three-level-phase-fraction prediction test. The test accuracy is wholly dependent upon the similarity between the simulated and real XRD patterns. Although the simulated XRD pattern was similar to the real version in general, a complete coincidence would not be possible. The object of our XRD pattern simulation was to develop an XRD pattern that appeared like those normally measured by ordinary lab XRD machines. For robustness, it is necessary to test the fully trained CNNs by employing XRD patterns measured by many other XRD machines. The fact that other real XRD data testing might have resulted in more mispredictions should not be disappointing, as data re-simulation with altered simulation conditions can sort out the problems. In addition, such a data re-preparation process has already been sufficiently customized, so that it would take only a day to prepare around a million XRD patterns.

In fact, none of the simulated patterns overlapped any real patterns. Although the ternary compositions were fixed as representative of 21 evenly distributed compositions when the Dataset_800k_org was prepared, the ternary composition for the real experimental data was completely randomly chosen and differed from the fixed compositions used for the Dataset_800k_org preparation. Of course, no composition overlap was also confirmed between the simulated and real data for both Dataset_800k_rand and Dataset_180k_rand. Despite the distinction between the simulated training dataset and the real test dataset, we would like to stress the 100% accuracy for the real dataset phase identification test, which validated the robustness of the suggested CNN approach. The main objective of the present approach was to identify real samples by using a CNN model trained with a simulated training dataset because of the lack of real experimental XRD data for training. It is extremely difficult and expensive to collect a sufficient amount of correctly labeled real experimental data in the materials research field. That is why most ML approaches^46^ were focused mainly on computational data created through density functional theory, molecular dynamics, thermodynamics, etc. The present investigation was motivated by the possibility that training with a huge amount of cheap synthetic data while testing with a small amount of expensive real experimental data could lead to a reliable prediction for real data.

As already mentioned in the Methods section, the simulated XRD patterns had many problems. The texture effect was ignored so that a sample with a preferred orientation would not have been appropriate for the test. Also, the particle size effect was not considered in the XRD simulation. Another absurdity for the simulated XRD data could be attributed to the fixed Lorentz and polarization factors according to the Bragg Brentano setting that is usually adopted in general lab XRD. Furthermore, the “real” datasets for testing are also synthetic mixtures in a sense since they would not include all the complexities of mixed phases. Basic bending of real pure sample XRD patterns would still be insufficient to simulate real-world samples, although they should be, of course, definitely superior to computer-simulated patterns.

One of the best ways to sort out actual data test degradation problems is to prepare a more generalized training dataset, the size of which should be considerably enhanced. The lab XRD patterns could exhibit a random distribution of peak overlap features caused by a variety of randomly chosen peak shapes. A substantial way to tackle the real data test degradation problem is to prepare a simulated dataset with a very sharp peak resolution, which is as good as high-resolution XRD, so that most calculated peaks are distinctive with no overlap. If we restricted all the real test data to only high-resolution data such as synchrotron source XRD data, the real data test accuracy would be definitely promising. Of course, high-resolution quality should also be adopted in the simulation. These sorts of further approaches are now in progress.

### Identification of new phases

Although the suggested CNNs would not work for the identification of novel unknown materials, it could be available to implicitly deduce the presence of novel materials in a mixture by considering the minimum cost (loss) function value for a mismatched sample. An unidentified sample does not have its correct label so that the cost function value cannot be evaluated but we assigned every possible label to this unidentified sample. For example, 8436 labels should be assigned to the unidentified ternary mixture samples one by one and their corresponding 8436 cost function values could be calculated, at which point a minimum cost function value could be pinpointed. This minimum cost function value for unknown ternary mixture samples constructs a novelty index. The higher the minimum cost function value the higher the possibility of novel phase inclusion.

We introduced two outsider compounds (BaO and CaO), which certainly reside outside the Sr-Li-Al-O quaternary compositional pool. They can be regarded as novel materials in the phase identification process. Several synthetic XRD patterns were generated by incorporating these compounds mixed with existing Al_2_O_3_ and SrO compounds. As a result, we generated two test datasets (BaO-SrO-Al_2_O_3_ and BaO-CaO-Al_2_O_3_), each of which includes ten random mixtures. Supplementary Fig. 4 shows the minimum cost function values (=novelty indexes) when each of the two outsiders were included in the mixture in comparison with the correctly matched dataset (Li_2_O-SrO-Al_2_O_3_).

The cost function value of the fully trained CNN_3 for a correctly matched dataset (Li_2_O-SrO-Al_2_O_3_) was around 10^−6^. On the contrary, the minimum cost function values for both the BaO-SrO-Al_2_O_3_ and BaO-CaO-Al_2_O_3_ test datasets was promoted on the order of 10^−3^ ~ 10^−1^, as evidenced in Supplementary Fig. 4. The minimum cost function values for the test dataset involving two outsiders simultaneously are higher than those with only an outsider. Although it is impossible for the phase identification CNN model to identify novel materials, it can judge at least whether a novel material is present in the mixture or not. This is far from complete but still deserves to be highlighted as a rough measure of novelty.

### Comparison with other baseline methods

We completed the phase identification using three well-known ML algorithms such as K-nearest Neighbor (KNN)^47^, Support Vector Machine (SVM)^48^, and Random Forrest (RF)^49^. Supplementary Table 2 shows the phase identification test accuracy for KNN, SVM, and RF based on the Dataset_180k_rand. We used hold-out test datasets for the test as was the CNN case. We did not fix a particular set of hyper-parameters but instead adopted several different hyper-parameters, which are described in Supplementary Table 2.

RF led to the best performance among the ML algorithms used. The RF result should therefore be considered as a baseline (87.63%). It should be, however, noted that all the test accuracies for KNN and SVM are far below the RF results and therefore do not deserve to be compared with the CNN result. In fact, the CNN test accuracy was nearly 100% for both the simulated and real data tests. The real data test was also completed for RF using the real Li_2_O-SrO-Al_2_O_3_ test dataset. Whereas our CNN model exhibited equivalent test accuracies both for the simulated and real data, the real data test accuracy for RF was conspicuously deteriorated by comparison with the simulated data test accuracy for RF as shown in Supplementary Table 2. The KNN and SVM results could be improved to a limited extent if hyper-parameters (e.g., number of neighbors, distance metric, tree depth, kernel, etc.) were changed. No matter what hyper-parameters were adopted, however, no remarkable improvement could be expected for KNN and SVM, as we have already tested many other sets of hyper-parameters. For instance, the adoption of dynamic time wrapping and Kullback–Leibler divergence as a distance metric for KNN made no difference in the test accuracy result. In addition, by retaining the same hyper-parameters, we executed a KNN classification with the well-known MNIST dataset and the promising test accuracies (95~97%) were obtained as shown in Supplementary Table 2. This would be good evidence for the validity of our hyper-parameter choice.

The disappointing test accuracy for KNN and SVM basically came from the relatively large number of classes (9177 classes) by comparison with the small size of the Dataset_180k_rand (183,521 samples). On average, there are only about 20 entries per class. This means that we have a serious data deficiency problem in the 4501-dimensional hyperspace, which led to the disappointing KNN and SVM test result. RF gave a much higher accuracy than KNN and SVM as the number of trees increased to 100. A single decision tree algorithm also gave a test accuracy similar to that of KNN and SVM. As the test accuracy for RF was saturated with respect to the number of trees, as clearly shown in Supplementary Table 2, a further increase in tree numbers would show no further improvement.

The three-hot-vector system we adopted for our CNN algorithm has greatly simplified the large classification problem. Such a simplification could be one of the reasons for the promising performance of the CNN algorithm. The remarkable outperformance of CNN over the other ML algorithms is surprising, but inexplicable. If the promising test result for CNN had been purely attributed to the information leakage the simpler KNN, SVM, and RF algorithms, which are known to be very well-established ML algorithms, should also have given us almost perfect test accuracy. As a matter of fact, however, the KNN, SVM, and RF actually led to test results that were a bit disappointing. The deep architecture of CNN seems to be responsible. More detailed discussions on the extreme performance difference between the CNN and the others is beyond the scope of the present investigation.

## Discussion

We developed CNN models that enable prompt phase identification as well as a rough measure of the relative fractions of the constituent phases in a multiphase inorganic mixture sample consisting of Sr, Li, Al, and O. We simulated XRD patterns for 170 inorganic compounds that could be synthesized from this quaternary compositional pool and finally prepared three XRD pattern datasets (Dataset_800k_org, Dataset_800k_rand, and Dataset_180k_rand) by combinatorically mixing them, and finally developed robust CNN models using these large datasets. The present data-driven approach was an unprecedented attempt that worked, as well as conventional rule-based XRD analysis tools. Although these results appear to be a small success accomplished in a limited compositional space, i.e., the Sr-Li-Al-O quaternary compositional pool, we verified the potential of the proposed deep-learning approach for use in XRD analysis. In addition, the present approach that was well established in a particular compositional system could be extended to any other compositional systems. We are confident that a reliable deep-learning-based XRD analyzer could be set up to work in any particular compositional system within a few weeks. For instance, if a Li-Co-Ni-Mn-O compositional system, which is very familiar to Li-battery researchers, was suggested, a promising robust XRD analysis tool could be in hand within a few weeks. We are waiting for such a suggestion and/or request. The limitation of our approach, however, is a so-called combinatorial explosion problem, so it cannot be applied to systems with high entropy.

The suggested deep-learning approach was motivated by a well-known problem wherein a rapid phase identification of mixture samples has been tricky to achieve both in academia and industry. Within this problem setting, the suggested method plays an auxiliary role for the traditional rule-based method (or another ML method) that finalizes the conventional, precise XRD analysis for an unknown sample, leading to an exact evaluation of structural parameters. We have never exaggerated the current status of the suggested deep-learning approach in comparison with other methods, but instead we focused on an efficient merge or combination between the suggested and conventional methods. It is certain that the suggested deep-learning approach enables rapid phase identification and a rough phase-fraction estimation that is a very good preliminary step to an ensuing, and more accurate, phase-fraction estimation, as well as to an exact evaluation of structural parameters by well-known Rietveld refinement^9^ or some other ML methods such as our previous symmetry identification, CNN^20^. In this regard, the suggested method would be useful for prompt phase identification in a relatively simple, well-known system. In addition, even real-time impurity identification in a continuous production line in an industrial setting could be available with the assistance of the suggested deep-learning approach.

## Methods

### Dataset preparation through XRD pattern simulations

The composition of interest was confined within Sr, Al, Li, and O, which are the typical elements of inorganic luminescent materials^37–39^. There were 174 ICSD entries in this compositional system. Five erroneous entries were precluded. Consequently, the remaining 169 entries were accounted for in the XRD pattern simulation. In particular, a recently discovered quaternary compound, Sr_2_LiAlO_4_, was also added, although this entry was not included in the 2018 version of the ICSD. Sr_2_LiAlO_4_ is known as a host material for Eu^2+^ activation that leads to promising luminescent material for use in LED applications^39^. As a result, we secured 170 entries in total, which were assigned to 38 classes. When all the duplicates were accounted for, 38 independent and unique structures remained. The 170 constituent compounds are listed along with their corresponding classes in Supplementary Table 3. The structure type and the lattice parameters for each of the 170 entries are also given in Supplementary Table 3. The CNN classification model includes 38 classes rather than 170 because of the many duplicated entries. For example, Al_2_O_3_ has 74 duplicates (variants). The duplicate entries are considered to have the same structure but with slightly different lattice sizes. Thus, the CNN classification model was trained to not distinguish duplicates belonging to the same class, as they constitute very similar XRD patterns despite the peak location shifts. The CNN was eventually trained such that many slightly different patterns could be recognized as an identical class. Some readers might misunderstand that our phase identification problem is a 38-class-classification problem, because our CNNs has 38 nodes at the output layer. However, the number of classes would no longer be 38, but we have 9177 classes for the simple phase identification and a much higher number of classes for the phase-fraction prediction. The number of categories (classes) is actually 9177 but only 38 output nodes in CNN were sufficient for such a complicated classification problem, as we adopted the so-called three-hot-vector system, which will be explained in detail below.

**Fig. 3:**
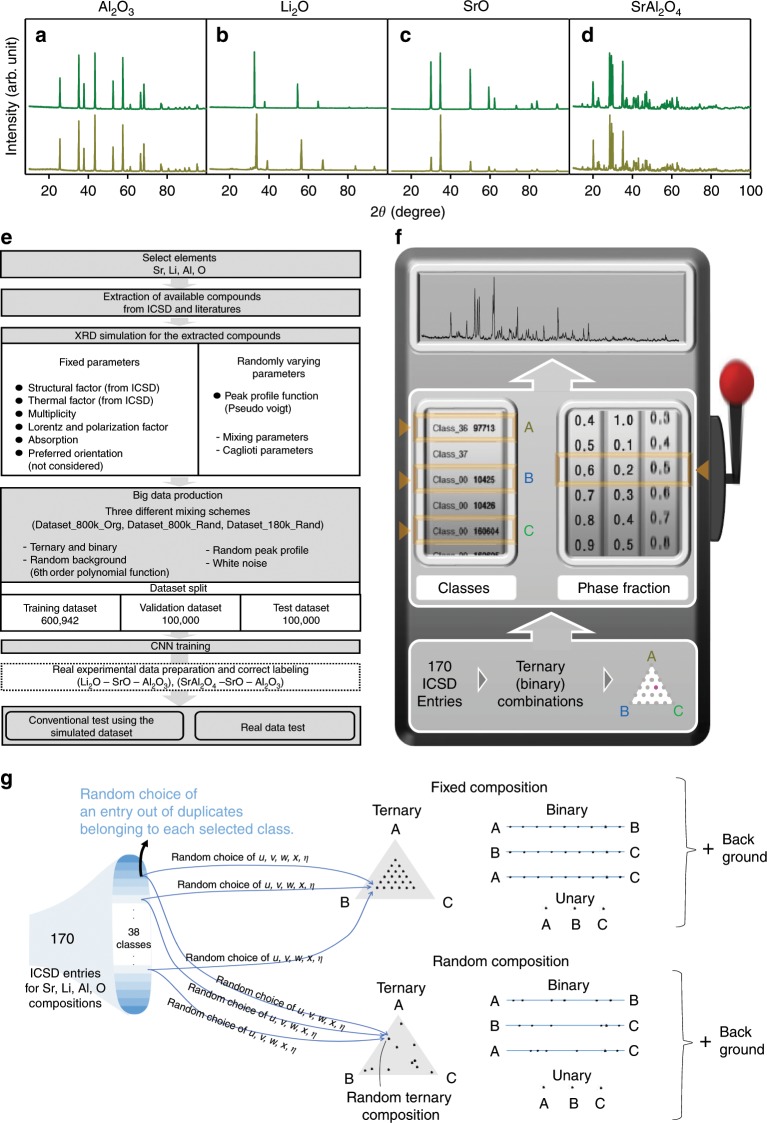
XRD data preparation protocol. Comparison between experimental and simulated XRD patterns for **a** Al_2_O_3_, **b** Li_2_O, **c** SrO, and **d** SrAl_2_O_4_. Green and brown lines stand for experimental and simulated XRD patterns, respectively. **e** A flow chart for the overall dataset preparation. **f** The brief scheme of constituent compound selection for the binary and ternary combinatorics. **g** A schematic for detailed mixture preparation procedures. There are two independent mixture composition determination protocols: fixed (evenly distributed) composition selection (upright) and random selection (downright). Each of these protocols leads to a total of _38_C_3_ × 21 + _38_C_2_ × 9 + _38_C_1_ (183,521) different mixtures (Dataset_180k_rand). To produce 800,942 mixtures (Dataset_800k_org and Dataset_800k_rand), stochastic repetitive choices are allowed for mixtures consisting of constituents with multiple variants (duplicates).

The powder XRD pattern simulation protocol is identical to the previous one used for symmetry identification of a CNN model^20^. The simulated XRD pattern preparation process involved such adjustable parameters as peak profile (Caglioti and mixing parameters), background, and white noise, in addition to the structural parameters and thermal factors obtained from the ICSD. We also adopted several fixed parameters such as multiplicity, polarization correction, and preferred orientation. Multiplicity can be obtained with ease from the symmetry data presented in the ICSD and polarization correction was applied to laboratory XRD using Bragg–Brantano geometry fitted with a graphite monochromator in the incident beam, and the preferred orientation was considered non-existent. We randomly assigned the adjustable parameters by referring to the plausible parameter values that typically appear in an ordinary lab-scale X-ray diffractometer. Figure 3a–d shows several simulated XRD patterns along with their corresponding experimental patterns. It should be impossible to differentiate whether these are synthetic or real using only the naked eye. In particular, a certain degree of texture (preferred orientation) could be observed in the case of experimental SrO XRD patterns (Fig. 3c), whereas no texture effect was taken into account in the simulation. Although this discrepancy could be eliminated by grinding the SrO powder samples for a while, we used the original SrO XRD data involving the texture as it is when we prepared the actual experimental test dataset. Despite such a discrepancy, we found that the phase identification accuracy turned out to be nearly 100% under all circumstances, as evidenced in the Results section. On the other hand, this sort of discrepancy might have led to the limited success of the phase-fraction prediction.

To begin with, we prepared 800,942 different XRD patterns for the first dataset by mixing binary or ternary components combinatorically chosen out of the simulated 170 entries. The dataset was randomly split into three parts to avoid overfitting: training (600,942 patterns), validation (100,000 patterns), and test (100,000 patterns) datasets. Every mixture sample in the dataset was composed of one, two, or three constituents, which was reasonable for the Sr-Li-Al ternary oxide composition pool when the Gibbs phase rule was considered. Every time we selected 2 or 3 compounds from the 170 entries to constitute ternary or binary combinatorics, the peak profile was randomly chosen so that nothing was the same among those 800,942 different XRD patterns. The ternary or binary combinatorics composed of entries belonging to the same class were all omitted. Also, randomly chosen background and white noise was applied to each mixture. The entire dataset preparation procedure is schematically described in Fig. 3e–g. In particular, Fig. 3g shows more details on the mixture composition assignment procedure along with the constituent selection for unary, binary, and ternary mixtures.

The ternary and binary mixtures were generated based on the combinatorics made of 38 classes, rather than 170. Then, we randomly assigned one of 170 duplicates (variants) to each constituent every time a class was selected. For example, we randomly assigned one out of 74 variants to Al_2_O_3_, when Al_2_O_3_ was selected as a constituent of a mixture of concern. As Al_2_O_3_ has many chances of appearing in many different mixtures, each of 74 variants appeared at least once. More of the dataset preparation details are described below.

The ternary mixture had 21 fixed compositions and 9 fixed compositions for the binary, for a total of _38_C_3_ × 21 + _38_C_2_ × 9 + _38_C_1_ (183,521) different mixtures. Simple mathematics might lead to quadruple repetitions per each mixture on average (i.e., 800,942/183,521 ≒ 4.36). However, the same mixtures (i.e., the same constituents and the same fractions) could be scarcely found in the entire dataset, because we adopted repetitive choices only for some mixtures with highly duplicated constituents. The rate of recurrence followed a reasonable principle whereby a mixture with the higher number of duplicates for each constituent would have more chances to be chosen. As a result, we eventually had 800,942 mixtures in total. Unless we adopted this reasonable data preparation protocol, many of the 74 Al_2_O_3_ variants (duplicates) would have far less of a chance to be chosen in comparison with Li_2_O_2_ with no variant, because each of the 38 classes would have exactly the same chance to be selected to constitute 183,521 distinct mixtures. As most of the classes have either no variant (duplicate), or only a few variants, according to this principle, the appearance of the same mixture would never be allowed to repeat.

Even mixtures with the same composition (i.e., the same constituent and the same phase fraction) have different peak positions due to the different lattice sizes originating from the different variants and also to the completely different peak shapes originating from the randomly chosen values of Caglioti (u, v, w) and mixing (η, x) parameters. It is evident that we adopted random u, v, w, x, and η values for each constituent and even different backgrounds and white noise, every time each of the 800,942 patterns was created. Consequently, whenever a certain compound was detected in many different mixtures, we would see a slightly different peak position (caused by the random selection out of many variants for the compound), a different peak profile, a different background, and a different form of white noise. We never recurrently used only the 38 constituents with fixed peak profiles during the mixture generation process. For example, Al_2_O_3_ appeared many times (around ~15,000 times) in 800,942 mixtures, but there was no chance to see the same pattern. Supplementary Fig. 5 shows two synthetic patterns, both of which represent Li_2_O-SrO-Al_2_O_3_ with a fraction of 0.38-0.24-0.38 and thereby our CNN model correctly identified these two patterns as a Li_2_O-SrO-Al_2_O_3_ mixture. Both the samples shared exactly the same constituent and the same fraction. As shown in Supplementary Fig. 5, however, they look completely different. Almost every peak position was conspicuously shifted due to the choice of different variants and the peak profile for all peaks were also different due to the random choice of u, v, w, x, and η values.

To rule out the similarity concern whereby many similar entries residing between the training and test datasets could have been responsible for the outstanding performance of CNNs, we prepared a completely different dataset, which adopted random ternary and binary compositions in contrast to the above-described dataset involving the fixed compositions for ternary and binary systems. It is certain that there must be absolutely no identical compositions in this case. Furthermore, in order to thoroughly rule out the data similarity concern, we prepared downsized dataset with random composition and with no repetitive selection, namely, the number of mixtures in this contracted dataset was reduced to _38_C_3_ × 21 + _38_C_2_ × 9 + _38_C_1_ (183,521). For the sake of convenience, three acronyms were assigned to the datasets described above, such that the original dataset with the even composition distribution is referred to as Dataset_800k_org, the same sized dataset with random compositions as Dataset_800k_rand, and the downsized dataset with random compositions as Dataset_180k_rand. The latter two auxiliary datasets were also randomly split into training, validation, and test datasets.

The relative fraction of constituent phases was pragmatically defined as the ratio between the maximum peak heights for constituent phases. This practically defined phase fraction will be referred to as a height fraction, which approximated neither the weight nor the molar fraction. However, the weight and molar fractions could be deduced from the height fraction, as there was a certain correlation between them, although it is impossible to derive a constitutive correlation formula due to the lack of data. Supplementary Table 4 compares the height fraction with the weight fraction and the mol. fraction, all of which were correctly measured using real experimental XRD pattern data for SrAl_2_O_4_-SrO-Al_2_O_3_ and Li_2_O-SrO-Al_2_O_3_ mixture systems. The terminology “phase fraction” in the present investigation designates the height fraction unless otherwise stated.

It is easy to evaluate the weight fraction from the blended XRD pattern by using a simple Rietveld refinement process. Supplementary Fig. 6 shows a full profile of the Rietveld refinement results for three simulated Li_2_O-SrO-Al_2_O_3_ mixtures with height fractions of 0.38-0.52-0.1, 0.52-0.38-0.1, and 0.66-0.24-0.1, which turned into real weight fractions of 0.09-0.67-0.23, 0.20-0.59-0.21, and 0.34-0.40-0.26, respectively. The results also show that the overall trend was consistent between the height and weight fractions. Once the height fraction was secured, there was no problem for the evaluation of the real important mixture fraction. If we collected more weight fraction data using the simulated mixtures on the top of already-secured experimental data given in Supplementary Table 4, a definite correlation formula could be obtained using a simple ML regression. However, that would be beyond the scope of the present investigation.

### CNN architecture

As CNN-based deep-learning techniques^10–19^ are now familiar in all science and engineering areas, a detailed description of CNN was not needed here; however, our previous report^20^ regarding the deep-learning technique for symmetry classification should clarify the present CNN approach for lay persons. Although a similar CNN approach was employed for both the previous and current studies, the main framework and the final goal thoroughly differed between the two approaches. The CNN architecture and training process for the present investigation was improved considerably by comparison with the previous case. For example, the dataset size was increased significantly, as 150,000 XRD patterns were used for the previous CNN for symmetry-classification CNN, but the current CNN model used 800,942 XRD patterns. In addition, unclean multiphase XRD patterns, which look much more realistic, were dealt with in the present investigation, whereas the previous case required neat and tidy single-phase XRD patterns.

**Fig. 4:**
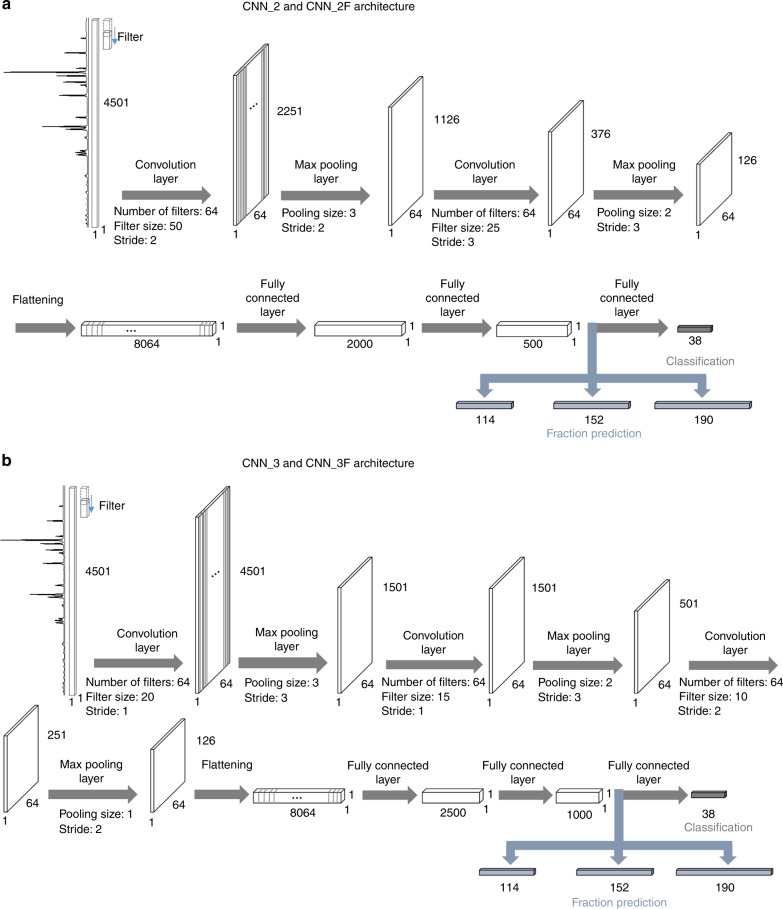
The schematic representation for CNN architectures. **a** CNN_2 and **b** CNN_3 architectures, which consisted of two and three convolution/max pooling layers, respectively. Three ensuing fully connected common layers followed. The number of filters, the kernel size, the pooling size, the stride, and the padding scheme are also given. The padding type was the “SAME” for all CNNs. Other deeper architectures such as CNN_4F, CNN_5F, and CNN_6F are schematically described in Supplementary Fig. 8.

CNN architectures consisting of several convolutional layers along with pooling layers and three ensuing fully connected layers were adopted for the phase identification. The version with two convolutional layers is referred to as CNN_2 and the other with three convolutional layers as CNN_3. These two representative versions of architecture are schematically described in Fig. 4 along with the numbers of filters, the kernel sizes, the pooling sizes, the strides, and the padding schemes. We adopted an awkward pooling (& stride) strategy wherein the stride is wider than the pooling size for one of the convolutional layers. In fact, this awkward pooling (& stride) strategy was adopted accidently during the hyper-parameter optimization process and thought of as just a feature dimension reduction doing no harm to the training, but it yielded a surprisingly faster convergence than the conventional pooling (& stride) scheme. Accordingly, we simply adopted such an unconventional pooling (and stride) strategy, although we knew that it appeared a bit awkward. We also tested a conventional pooling (& stride) strategy with strides that were equal to the pooling size for both CNN_2 and CNN_3. As shown in Supplementary Fig. 7, the convergence for the unconventional pooling (& stride) setting was much faster than the conventional one at the early stage of training, although the final performance was almost the same in later epochs, namely, nearly 100% test accuracy was secured in the end for both settings. Accordingly, it was revealed that the pooling (& stride) strategy adopted in the present investigation did not impair the CNN performance despite the unconventionality.

A rectified linear unit was adopted as an activation function for the convolutional layer and a simple linear function for the fully connected layers. A dropout was implemented only for the fully connected layers. The final activation function for the last fully connected layer was a sigmoid function and the ensuing cost (or loss) function was a cross-entropy function. The input featured a 4501 × 1 vector shape and the output featured a 38 × 1, 114 × 1, 156 × 1, and 196 × 1 vector shape. As the number of constituent phases in a mixture was confined to no more than three, the output vector was expressed as a sum of three one-hot-vectors in 38 × 1, 114 × 1, 156 × 1, and 196 × 1 shapes, which we referred to as a three-hot-vector. Consequently, it was not possible to use the softmax activation function and, instead, a sigmoid activation function was adopted as the three-hot-vector was employed in the present case. We used an Adam optimizer. The running rate was fixed at 0.001 for every epoch for the phase identification, but we reduced it to 0.0001 only in the last epoch for the three-level-phase-fraction prediction. The training/validation accuracy was defined as a percentage of correct matching between the CNN model prediction and the ground truth, which was averaged for a batch that included 1000 XRD patterns. Of course, many attempts involved other architectures, but we concluded that the architectures involving two or three convolutional layers outperformed the others for phase identification and a CNN architecture with a larger number of convolution layers outperformed those with fewer convolution layers for phase-fraction prediction.

## Supplementary information

Supplementary Information

Description of Additional Supplementary Files

Supplementary Data 1

Supplementary Data 2

Supplementary Software 1

Supplementary Software 2

## Data Availability

All data generated or analyzed during this study are included in this published article (and its Supplementary Information files) and the datasets used for CNNs during the current study are available from the corresponding author on reasonable request.

## Code availability

The codes used for this study and the fully trained weights are included in the Supplementary Software and Dataset.
