# Publisher Correction: A deep-learning technique for phase identification in multiphase inorganic compounds using synthetic XRD powder patterns

**DOI:** 10.1038/s41467-020-14512-9

**Published:** 2020-01-30

**Authors:** Jin-Woong Lee, Woon Bae Park, Jin Hee Lee, Satendra Pal Singh, Kee-Sun Sohn

**Affiliations:** Faculty of Nanotechnology and Advanced Materials Engineering, Sejong University, Seoul, 143-747 Republic of Korea

**Keywords:** X-ray diffraction, Inorganic chemistry, Computational methods

Correction to: *Nature Communications* 10.1038/s41467-019-13749-3, published online 03 January 2020.

The original version of this Article contained an error in the second sentence of the Abstract, which incorrectly read ‘We simulate plausible powder X-ray powder diffraction (XRD) patterns.’ The correct version removes ‘powder’ before ‘diffraction’.

The original version of this Article contained an error in the first sentence of the Introduction, which incorrectly read ‘We have recently discovered many novel inorganic functional materials by employing powder X-ray powder diffraction (XRD) analysis.’ The correct version removes ‘powder’ before ‘diffraction’.

The original version of this article also contained an error in the last sentence of the third paragraph of the ‘Real data test’ section of the Results, which incorrectly read ‘Basic bending of real pure sample XRD patterns would still be insufficient to simulate real-world samples, although they should be, of course, definitely superior to computer-simulated patterns.’ The correct version states ‘blending’ in place of ‘bending’.

These have been corrected in both the PDF and HTML versions of the Article.

