# Supplementary material for: A deep-learning technique for phase identification in multiphase inorganic compounds using synthetic XRD powder patterns
Source: Nat Commun. 2020 Jan 3;11:86. doi: 10.1038/s41467-019-13749-3 (PMC6941984; doi:10.1038/s41467-019-13749-3)
Supplement: Supplementary file 1 — Supplementary Information [file 41467_2019_13749_MOESM1_ESM.pdf]

Supplementary Information

A deep-learning technique for phase identification in multiphase inorganic compounds using synthetic XRD powder patterns

Lee et al.

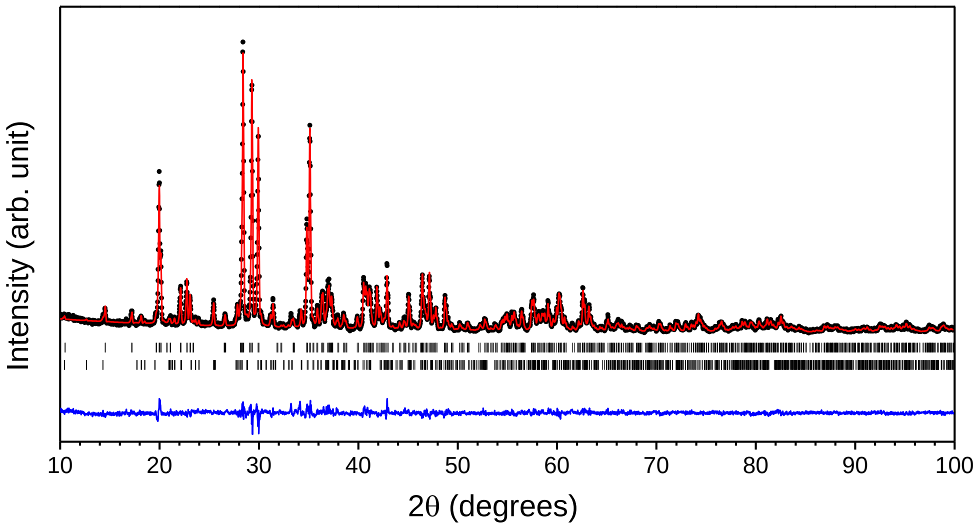

Lattice parameters and the agreement factors obtained after the Two-Phase-Rietveld refinement for $SrAl_2O_4$ and $Sr_4Al_{14}O_{25}$.

$SrAl_2O_4$ Phase:
Space group: $P2_1$
Number of formula per unit cell (Z): 2
Lattice parameters: a = 8.4439 (3), b = 8.8208(3), c = 5.1591(2) Å, α = γ = 90°, β = 93.409(2)°
Phase fraction: 85%
$Sr_4Al_{14}O_{25}$ Phase:
Space group: Pmma
Number of formula per unit cell (Z): 2
Lattice parameters: a = 24.784(2), b = 8.4808(8), c = 4.862(6) Å, α = β = γ = 90°
Phase fraction:15%
Agreement factors: R_p = 14.0, R_{wp} = 14.8, R_{exp} = 11.17, χ^2 = 1.76

Supplementary Figure 1 A full pattern fit obtained after using two phase Rietveld refinement on powder x-ray diffraction data in the 2θ range 10-100° with monoclinic structure in the $P2_1$ space group for $SrAl_2O_4$ phase and orthorhombic structure in the Pmma space group for $Sr_4Al_{14}O_{25}$ phase. Black dots, red line and blue line represents the experimental, calculated and difference profiles, respectively. The vertical tick marks above the difference profile in first and second row from top denotes the position of Bragg reflections for $SrAl_2O_4$ and $Sr_4Al_{14}O_{25}$ phases, respectively.

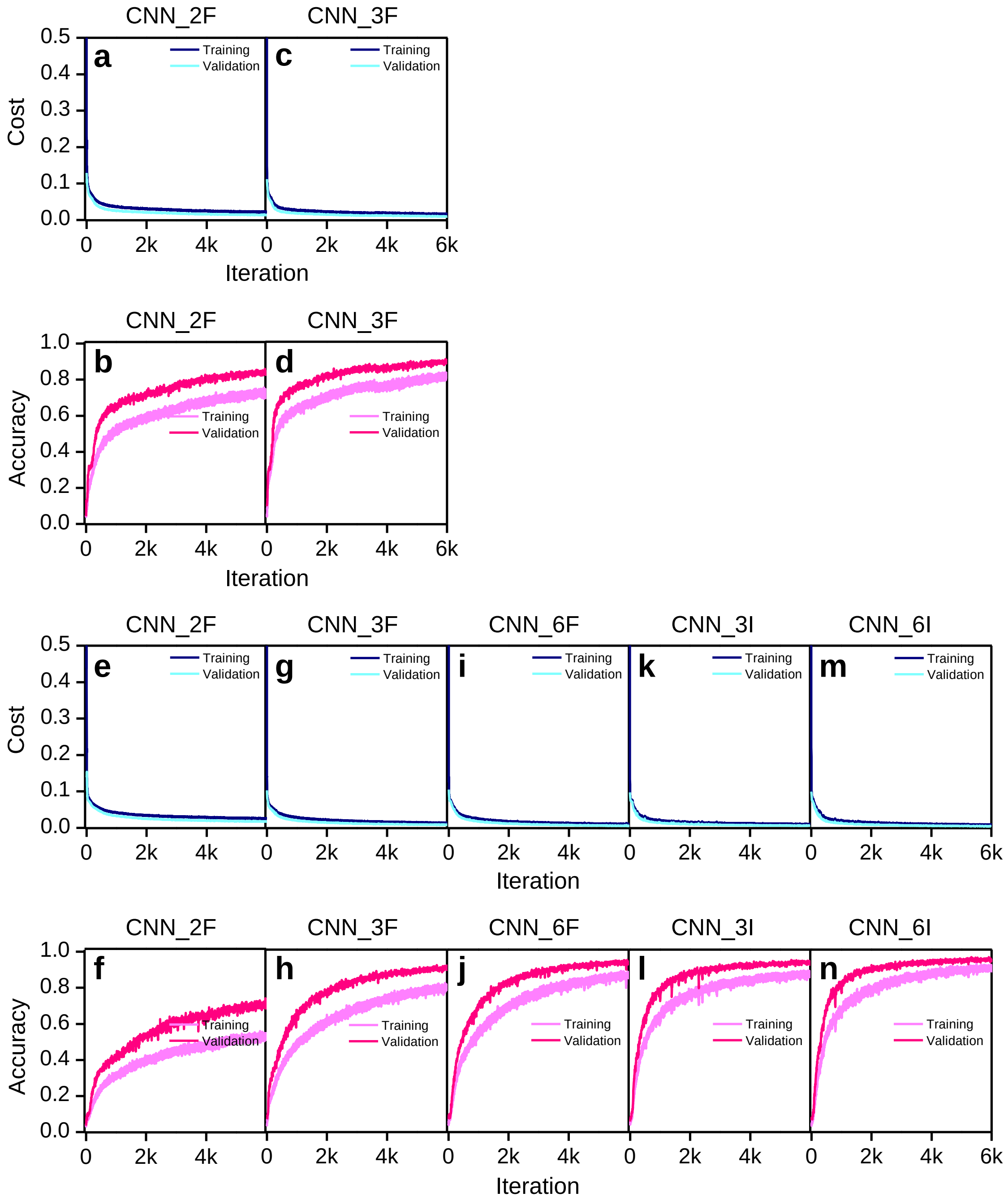

Supplementary Figure 2 The training loss/accuracy and the validation loss/accuracy for the four- and five-level-phase-fraction predictions plotted as a function of the iteration number up to the 10th epoch. The four-level-phase-fraction predictions are plotted only for CNN_2F (**a** and **b**) and CNN_3F (**c** and **d**) architectures. Additional five-level-phase-fraction predictions based on CNN_2F (**e** and **f**) and CNN_3F (**g** and **h**) CNN_6F (**i** and **j**), CNN_3I (**k** and **l**) and CNN_6I (**m** and **n**) are also given.

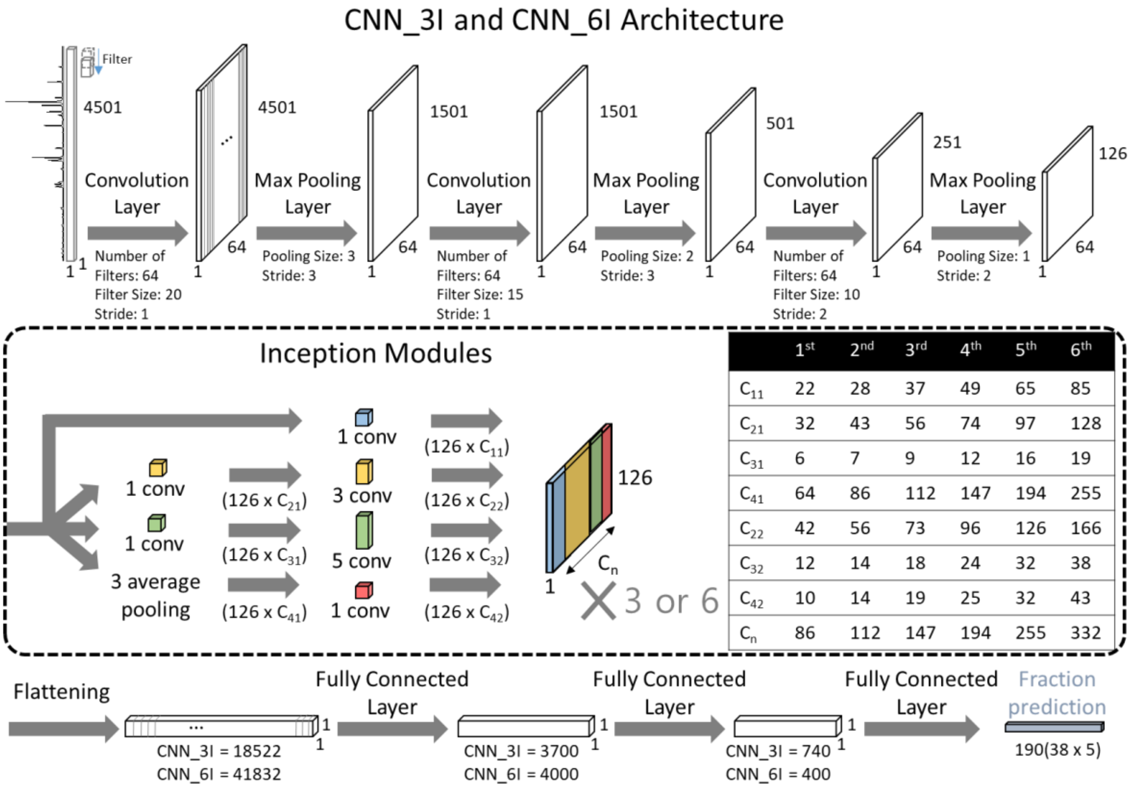

Supplementary Figure 3 The inception net used for the five-level-phase-fraction prediction (CNN_3I, and CNN_6I). Typical 1D convolutional layers adopted CNN_3 and 3F (top row), the inception module along with the number of kernels listed in the table on the right side (middle row,) and the typical fully connected layers (bottom row). Three or six inception modules that are actually attached in sequence in the middle row were omitted but can be simply represented as 'x 3' or 'x 6'.

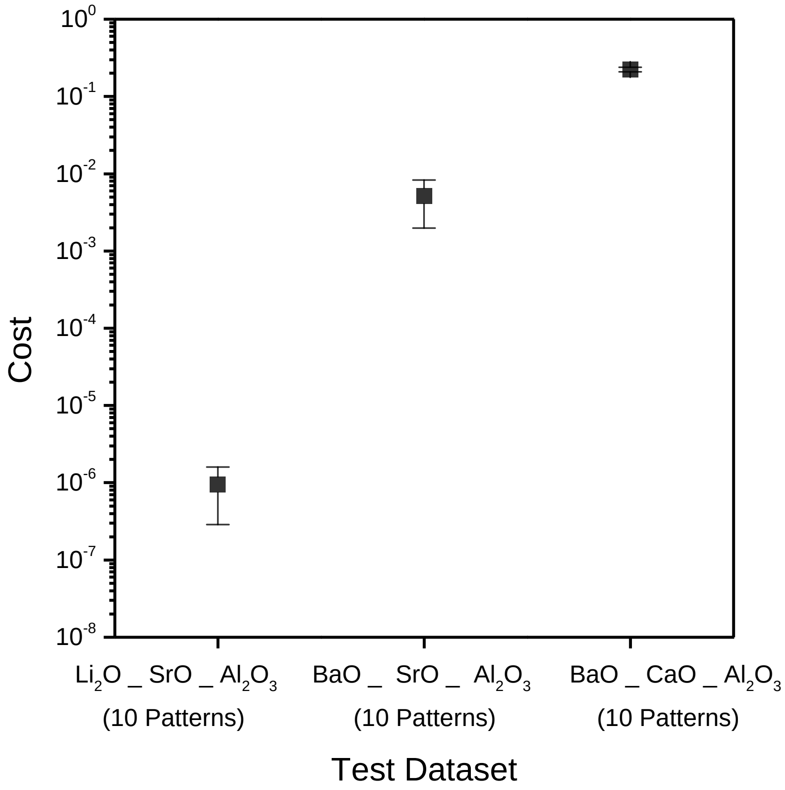

Supplementary Figure 4 The minimum cost (loss) function values for two test datasets (BaO-SrO-Al_2O_3 and BaO-CaO-Al_2O_3) wherein the two outsiders were included in the mixture in comparison with the correctly matched dataset (Li_2O-SrO-Al_2O_3). The square dots and whiskers indicate the mean and standard deviation.

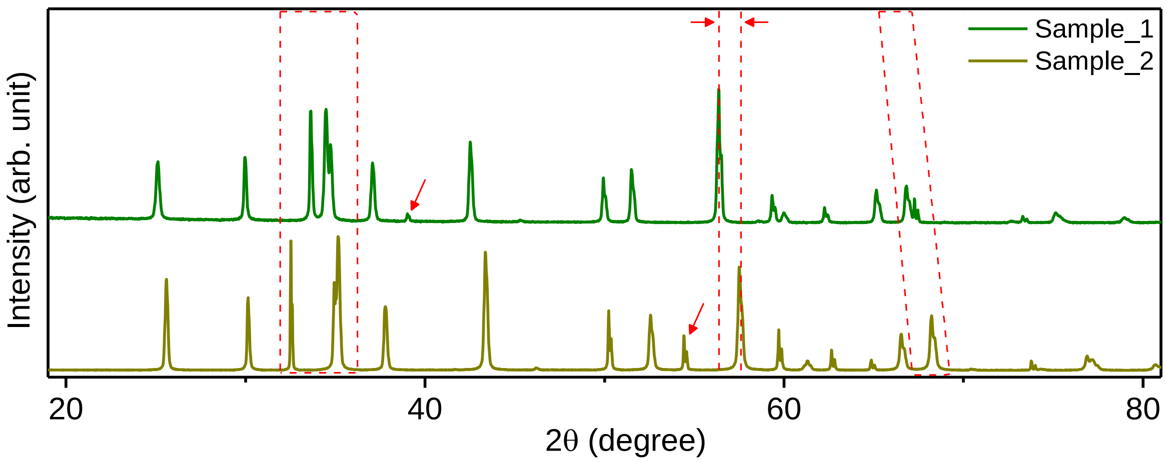

Supplementary Figure 5 Two synthetic patterns, both of which represent Li_2O-SrO-Al_2O_3 with a fraction of 0.38-0.24-0.38, and thereby our CNN model correctly identified these two patterns as a Li_2O-SrO-Al_2O_3 mixture. Both the sample shared exactly the same constituent and the same fraction. However, they look completely different. It is clearly observed that almost every peak position was conspicuously shifted due to the choice of different variants, and the peak profile for all peaks are also different due to the random choice of u, v, w, x, and η values. The conspicuous difference is marked by dashed lines and arrows.

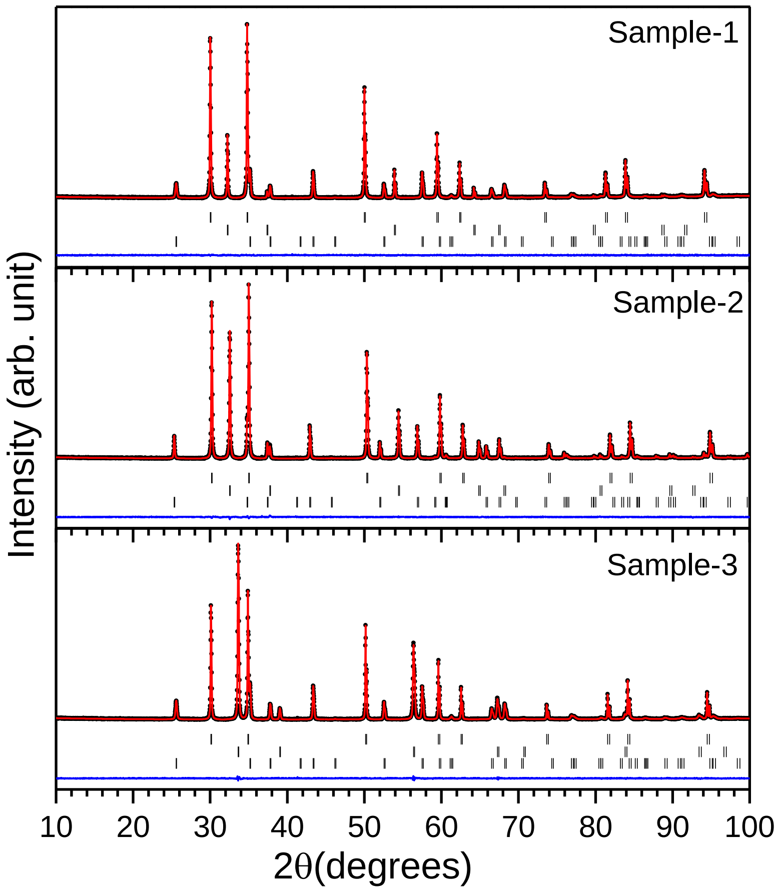

Height and weight fraction (%) of Li_2O, SrO and Al_2O_3 in three different samples

Sample No.	Li_2O		SrO		Al_2O_3	
	Height fraction	Wt. fraction	Height fraction	Wt. fraction	Height fraction	Wt. fraction
Sample-1	38%	9.42%	52%	67.46%	10%	23.11 %
Sample-2	52%	20.27%	38%	58.50%	10%	21.23 %
Sample-3	66%	33.79%	24%	40.05%	10%	26.16 %

Supplementary Figure 6 Rietveld refinement fit for three different samples containing a mixture of SrO ($Fm\bar{3}m$), Li_2O ($Fm\bar{3}m$) and Al_2O_3 ($R\bar{3}c$) in different proportions. Black dots, red line, blue line in the figure represents the simulated, calculated and difference profiles, respectively. The vertical tick marks above the difference profile in the first, second and third lines from the top represents the Bragg's position for SrO, Li_2O and Al_2O_3, respectively.

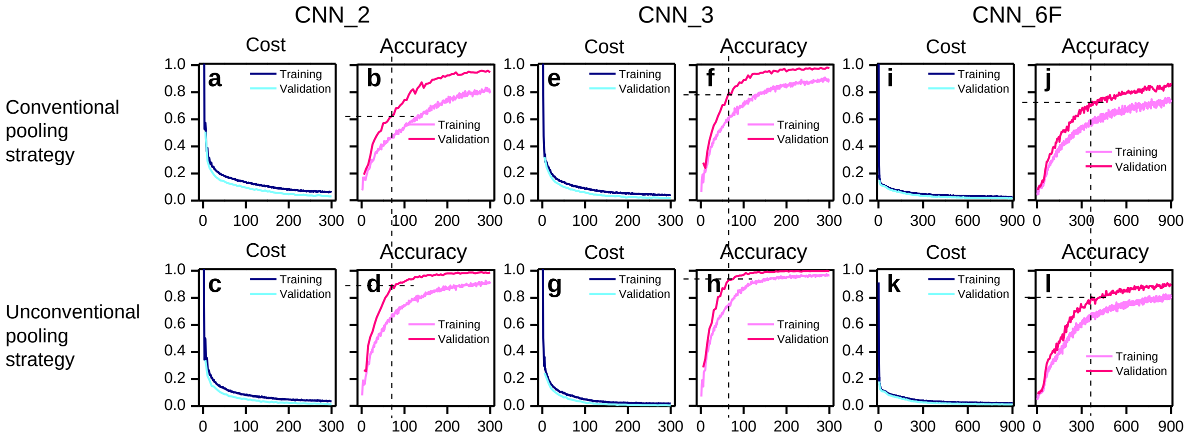

Supplementary Figure 7 The training loss/accuracy and the validation loss/accuracy at the early stage of training for the conventional and unconventional pooling strategies in the phase identification based on CNN_2 (**a**, **b**, **c**, and **d**) and CNN_3 (**e**, **f**, **g**, and **h**), and the three-level-phase-fraction prediction based on CNN_6F (**i**, **j**, **k**, and **l**). The intersect on the vertical dashed line clearly indicates the faster training for the unconventional pooling strategy. The conventional pooling (& stride) strategy stands for a stride that is greater than the pooling size and the unconventional strategy for the stride is equal to the pooling size.

CNN_4F Architecture

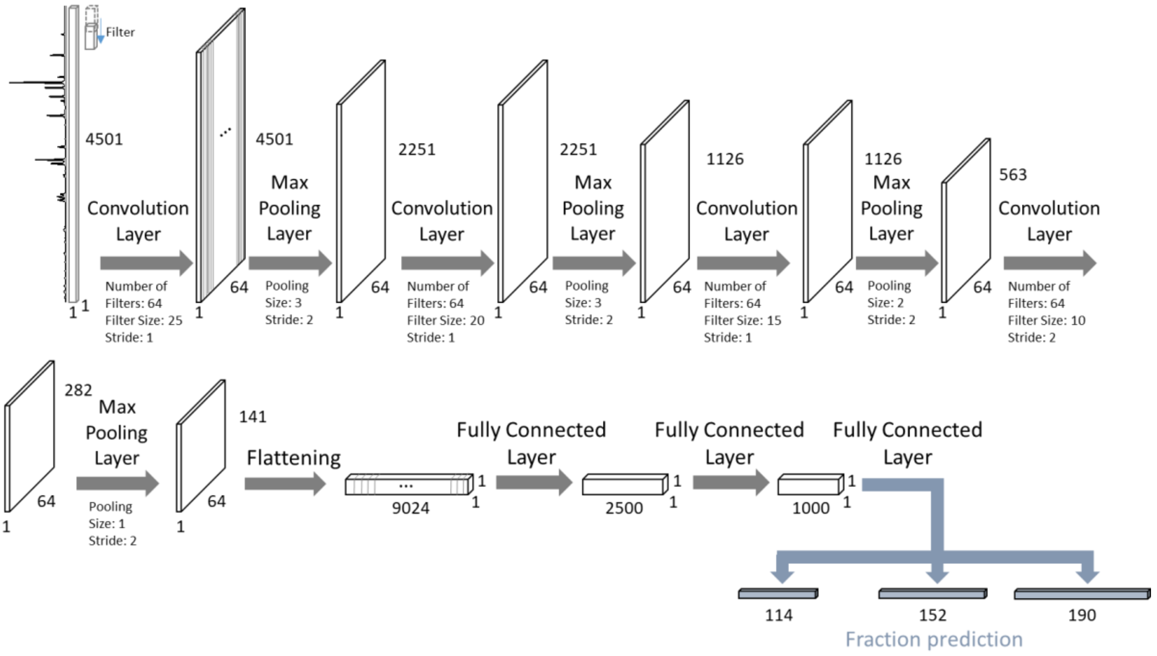

CNN_5F Architecture

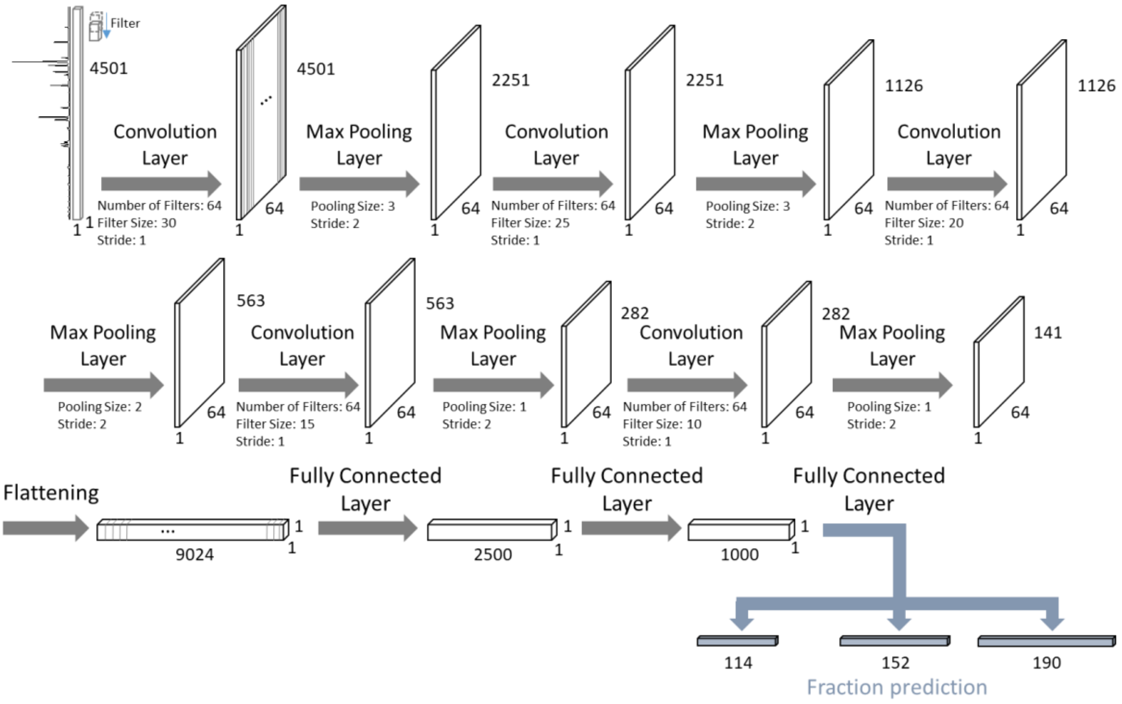

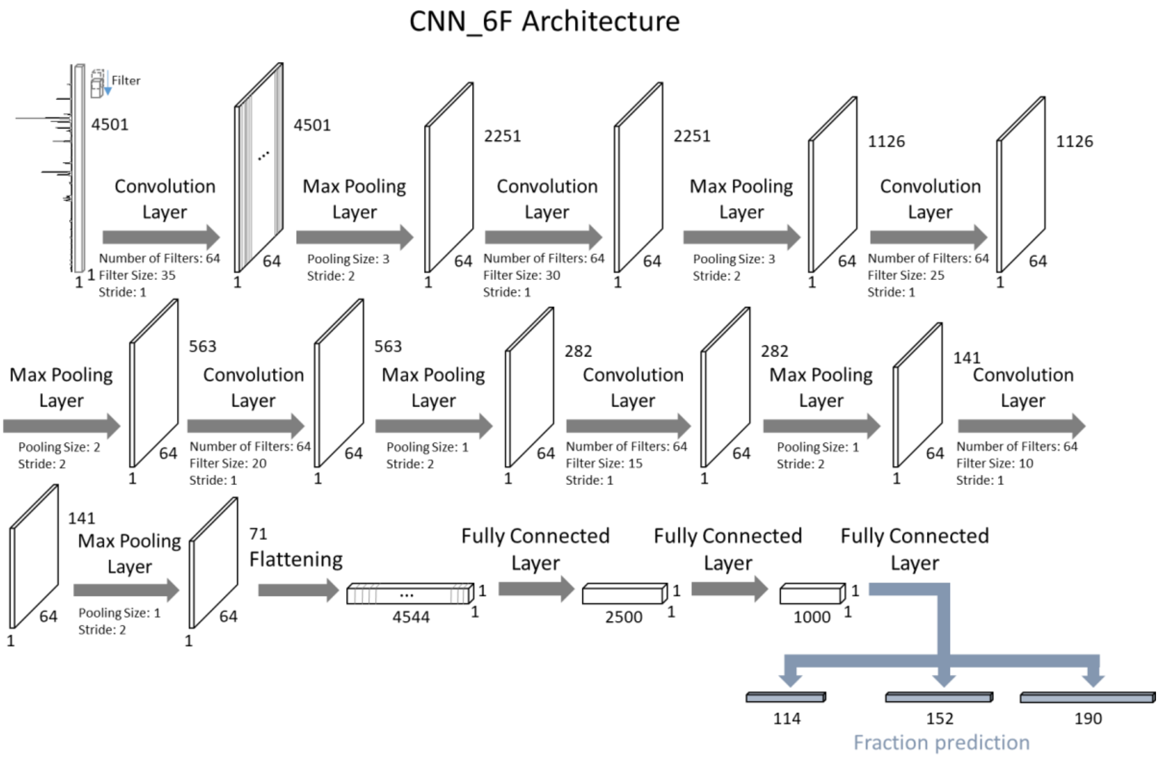

Supplementary Figure 8 The schematic representation for CNN_4F, 5F, and 6F architectures.

Four- and Five-Step-Phase-Fraction Prediction Test Result

		CNN_2F	CNN_3F	CNN_6F	CNN_3I	CNN_6I
4_level_fraction_ prediction (10 Epochs)	Simulated XRD test dataset 100,000 patterns	87.29%	91.43%	–	–	–
5_level_fraction_ prediction (10 Epochs)	Simulated XRD test dataset 100,000 patterns	77.65%	92.44%	94.76%	94.28%	95.90%

Supplementary Table 1 The test results from the four- and five-level-phase-fraction predictions for the simulated test datasets.

Baseline Method Result

KNN Test Accuracy: (Dataset_180k_rand)
35.72% (k=3, Euclidean), 32.39% (k=5, Euclidean), 29.84% (k=13, Euclidean), 25.57% (k=51, Euclidean)
**SVM Test Accuracy: (Dataset_180k_rand)**
45.76% (radial basis function kernel)
**RF Test Accuracy: (Dataset_180k_rand)**
69.37% (# of trees=5), 78.47% (# of trees=10), 81.48% (# of trees=15), 86.34% (# of trees=50), 87.63% (# of trees=100)
**RF Real Data Test Accuracy: (The real test dataset for Li_2O-SrO-Al_2O_3 ternary mixtures)**
20% (# of trees=5), 28% (# of trees=10), 34% (# of trees=15), 42% (# of trees=50), 58% (# of trees=100)

KNN Test Accuracy: (MNIST dataset)
97.05% (k=3, Euclidean), 96.88% (k=5, Euclidean), 96.53% (k=13, Euclidean), 95.33% (k=51, Euclidean)

Supplementary Table 2 The KNN, SVM and RF test results from the phase identification. Some important hyper-parameters are presented in the parenthesis along with the test accuracy. The real data test accuracies for RF are only presented but those for the others are omitted since they are too low. The KNN results for the MNIST data classification are presented on the bottom. The hyper-parameters used for the Dataset_180k_rand are identical to those for the MNIST data.

Supplementary Table 3 The list of 170 constituent compounds. The structure type and the number of duplicates for each of 38 classes are also given.

38 Classes	ICSD_Num	Compound	Space Group	Lattice Parameter					
				a	b	c	α	β	γ
Corundum-Al_2O_3 (Al-O) Class_Num_0	10425	Al_2O_3	R-3cH (167)	4.754(1)	4.754(1)	12.99(2)	90	90	120
	10426	Al_2O_3	R-3cH (167)	4.844(2)	4.844(2)	13.27(2)	90	90	120
	160604	Al_2O_3	R-3cH (167)	4.7617(1)	4.7617(1)	12.9990(2)	90	90	120
	160605	Al_2O_3	R-3cH (167)	4.7698(1)	4.7698(1)	13.0243(4)	90	90	120
	160606	Al_2O_3	R-3cH (167)	4.7805(1)	4.7805(1)	13.0561(3)	90	90	120
	160607	Al_2O_3	R-3cH (167)	4.7936(1)	4.7936(1)	13.0955(3)	90	90	120
	160901	Al_2O_3	R-3cH (167)	4.7598(1)	4.7598(1)	12.9943(1)	90	90	120
	160902	Al_2O_3	R-3cH (167)	4.761(1)	4.761(1)	12.997(3)	90	90	120
	160903	Al_2O_3	R-3cH (167)	4.761(1)	4.761(1)	12.997(3)	90	90	120
	160904	Al_2O_3	R-3cH (167)	4.761(1)	4.761(1)	12.999(1)	90	90	120
	164617	Al_2O_3	R-3cH (167)	4.7600(1)	4.7600(1)	12.9943(3)	90	90	120
	165594	Al_2O_3	R-3cH (167)	4.7591162(9)	4.7591162(9)	12.99731160(12)	90	90	120
	197685	Al_2O_3	R-3cH (167)	4.758	4.758	12.991	90	90	120
	198101	Al_2O_3	R-3cH (167)	4.7576	4.7576	12.9834	90	90	120
	24005	Al_2O_3	R-3cR (167)	5.12(1)	5.12(1)	5.12(1)	55.28	55.28	55.28
	24851	Al_2O_3	R-3cH (167)	4.763	4.763	13	90	90	120
	25778	Al_2O_3	R-3cH (167)	4.7589(10)	4.7589(10)	12.991(5)	90	90	120
	26790	Al_2O_3	R-3cR (167)	5.128	5.128	5.128	55.27	55.27	55.27
	30024	Al_2O_3	R-3cH (167)	4.7657(9)	4.7657(9)	13.010(14)	90	90	120
	30025	Al_2O_3	R-3cH (167)	4.7517(9)	4.7517(9)	12.965(17)	90	90	120
	30026	Al_2O_3	R-3cH (167)	4.7418(10)	4.7418(10)	12.921(20)	90	90	120
	30027	Al_2O_3	R-3cH (167)	4.7351(9)	4.7351(9)	12.901(17)	90	90	120
	30028	Al_2O_3	R-3cH (167)	4.7242(10)	4.7242(10)	12.881(18)	90	90	120
	30029	Al_2O_3	R-3cH (167)	4.7212(7)	4.7212(7)	12.872(14)	90	90	120
	30030	Al_2O_3	R-3cH (167)	4.7145(10)	4.7145(10)	12.851(22)	90	90	120
	31545	Al_2O_3	R-3cH (167)	4.7640(1)	4.7640(1)	13.0091(3)	90	90	120

	31546	Al_2O_3	R-3cH (167)	4.75860(2)	4.75860(2)	12.9906(1)	90	90	120
	31547	Al_2O_3	R-3cH (167)	4.75860(2)	4.75860(2)	12.9906(1)	90	90	120
	31548	Al_2O_3	R-3cH (167)	4.7640(1)	4.7640(1)	13.0091(3)	90	90	120
	33639	Al_2O_3	R-3cR (167)	5.13(2)	5.13(2)	5.13(2)	55.27(8)	55.27(8)	55.27(8)
	51687	Al_2O_3	R-3cH (167)	4.7597(1)	4.7597(1)	12.9935(3)	90	90	120
	52024	Al_2O_3	R-3cH (167)	4.7570(6)	4.757	12.9877(35)	90	90	120
	52044	Al_2O_3	R-3cH (167)	4.7570(6)	4.757	12.9877(35)	90	90	120
	52647	Al_2O_3	R-3cH (167)	4.758	4.758	12.99	90	90	120
	52648	Al_2O_3	R-3cH (167)	4.7602(4)	4.7602	12.9933(17)	90	90	120
	56085	Al_2O_3	R-3cR (167)	5.12(1)	5.12(1)	5.12(1)	55.28	55.28	55.28
	600672	Al_2O_3	R-3cH (167)	4.7591(5)	4.7591(5)	12.9877(13)	90	90	120
	60419	Al_2O_3	R-3cH (167)	4.7606(5)	4.7606(5)	12.994(1)	90	90	120
	608993	Al_2O_3	R-3cH (167)	4.803	4.803	13.13	90	90	120
	608994	Al_2O_3	R-3cH (167)	4.813	4.813	13.15	90	90	120
	608995	Al_2O_3	R-3cH (167)	4.822	4.822	13.17	90	90	120
	608996	Al_2O_3	R-3cH (167)	4.832	4.832	13.18	90	90	120
	608997	Al_2O_3	R-3cH (167)	4.844	4.844	13.24	90	90	120
	608998	Al_2O_3	R-3cH (167)	4.847	4.847	13.25	90	90	120
	609001	Al_2O_3	R-3cH (167)	4.7582	4.7582	12.991	90	90	120
	609004	Al_2O_3	R-3cH (167)	4.758	4.758	12.991	90	90	120
	63647	Al_2O_3	R-3cH (167)	4.7586(1)	4.7586(1)	12.9897(1)	90	90	120
	63648	Al_2O_3	R-3cH (167)	4.7586(1)	4.7586(1)	12.9897(1)	90	90	120
	64713	Al_2O_3	R-3cH (167)	4.718(6)	4.718(6)	12.818(14)	90	90	120
	68591	Al_2O_3	R-3cH (167)	4.76050(5)	4.76050(5)	12.9956(2)	90	90	120
	73076	Al_2O_3	R-3cH (167)	4.76	4.76	12.993	90	90	120
	73724	Al_2O_3	R-3cH (167)	4.7540(5)	4.7540(5)	12.9820(6)	90	90	120
	73725	Al_2O_3	R-3cH (167)	4.7540(5)	4.7540(5)	12.9820(6)	90	90	120
	75479	Al_2O_3	R-3cH (167)	4.7554(3)	4.7554(3)	12.9910(6)	90	90	120
	75559	Al_2O_3	R-3cH (167)	4.7589(4)	4.7589(4)	12.9919(3)	90	90	120
	75560	Al_2O_3	R-3cH (167)	4.7589(4)	4.7589(4)	12.9919(3)	90	90	120
	77810	Al_2O_3	R-3cH (167)	4.7598(1)	4.7598	12.9924(3)	90	90	120
	85137	Al_2O_3	R-3cH (167)	4.7607(7)	4.7607(7)	12.997(2)	90	90	120

	88027	Al_2O_3	R-3cH (167)	4.7589(1)	4.7589(1)	12.991(1)	90	90	120
	88028	Al_2O_3	R-3cH (167)	4.7585(2)	4.7585(2)	12.990(1)	90	90	120
	88029	Al_2O_3	R-3cH (167)	4.7597(1)	4.7597(1)	12.993(1)	90	90	120
	89662	Al_2O_3	R-3cH (167)	4.649(6)	4.649(6)	12.687(8)	90	90	120
	89663	Al_2O_3	R-3cH (167)	4.641(7)	4.641(7)	12.666(8)	90	90	120
	89664	Al_2O_3	R-3cH (167)	4.625(10)	4.625(10)	12.645(13)	90	90	120
	89665	Al_2O_3	R-3cH (167)	4.62(3)	4.62(3)	12.57(5)	90	90	120
	92630	Al_2O_3	R-3cH (167)	4.7602(4)	4.7602(4)	12.993(2)	90	90	120
	92631	Al_2O_3	R-3cH (167)	4.7602(4)	4.7602(4)	12.993(2)	90	90	120
	93096	Al_2O_3	R-3cH (167)	4.7599(5)	4.7599(5)	12.994(2)	90	90	120
	9770	Al_2O_3	R-3cH (167)	4.7607(9)	4.7607(9)	12.9947(17)	90	90	120
	9771	Al_2O_3	R-3cH (167)	4.7538(6)	4.7538(6)	12.9725(5)	90	90	120
	9772	Al_2O_3	R-3cH (167)	4.7474(4)	4.7474(4)	12.9542(5)	90	90	120
	9773	Al_2O_3	R-3cH (167)	4.7437(5)	4.7437(5)	12.9430(5)	90	90	120
	9774	Al_2O_3	R-3cH (167)	4.7406(5)	4.7406(5)	12.9326(16)	90	90	120
	9775	Al_2O_3	R-3cH (167)	4.7352(7)	4.7352(7)	12.9176(11)	90	90	120
Spinel-defect (Al-O) Class_Num_1	249140	$Al_{2.67}O_4$	Fd-3mZ (227)	7.93820(10)	7.93820(10)	7.93820(10)	90	90	90
	28260	$(Al_2O_3)_{5.3333}$	Fd-3mS (227)	7.906	7.906	7.906	90	90	90
	39014	$(Al_2O_3)_{1.333}$	Fd-3mS (227)	7.906	7.906	7.906	90	90	90
	39104	$Al_{2.66}O_4$	Fd-3mS (227)	7.906	7.906	7.906	90	90	90
	603780	$Al_{2.67}O_4$	Fd-3mZ (227)	7.947(10)	7.947(10)	7.947(10)	90	90	90
	66558	$Al_{2.144}O_{3.2}$	Fd-3mZ (227)	7.914(2)	7.914(2)	7.914(2)	90	90	90
	66559	$Al_{2.144}O_{3.2}$	Fd-3mZ (227)	7.911(2)	7.911(2)	7.911(2)	90	90	90
	68770	$Al_{21.333}O_{32}$	Fd-3mS (227)	7.9056(4)	7.9056(4)	7.9056(4)	90	90	90
	69213	$Al_{2.667}O_4$	Fd-3mS (227)	7.948(2)	7.948(2)	7.948(2)	90	90	90
$AlFeO_3$ (Al-O) Class_Num_2	247304	Al_2O_3	$Pna2_1$ (33)	4.8437	8.33	8.9547	90	90	90
	84375	Al_2O_3	$Pna2_1$ (33)	4.8437(2)	8.3300(3)	8.9547(4)	90	90	90
	94485	Al_2O_3	$Pna2_1$ (33)	4.8340(1)	8.3096(2)	8.9353(2)	90	90	90
Ga_2O_3 (Al-O) Class_Num_3	66560	$Al_{2.427}O_{3.64}$	C12/m1 (12)	11.854(5)	2.904(1)	5.622(2)	90	103.83(7)	90
	82504	Al_2O_3	C12/m1 (12)	11.795(5)	2.91(1)	5.621(7)	90	103.79	90
gamma (Al-O) Class_Num_4	68771	$Al_{10.666}O_{16}$	$I4_1$/amdS (141)	5.600(2)	5.600(2)	7.854(6)	90	90	90
	99836	$(Al_2O_3)_{1.333}$	$I4_1$/amdZ (141)	5.652(1)	5.652(1)	7.871(5)	90	90	90

$AgSbO_3$ (Al-O) Class_Num_5	291495	Al_2O_3	Fd-3mZ (227)	7.94	7.94	7.94	90	90	90
$Cr_2Mg_4O_4$ (Al-O) Class_Num_6	30267	$(Al_2O_3)_{1.333}$	Fm-3m (225)	3.95	3.95	3.95	90	90	90
Fluorite-CaF_2 (Al-O) Class_Num_7	28919	Al_2O	Fm-3m (225)	4.98	4.98	4.98	90	90	90
NaCl (Al-O) Class_Num_8	28920	AlO	Fm-3m (225)	5.67	5.67	5.67	90	90	90
Rh_2S_3 (Al-O) Class_Num_9	151590	Al_2O_3	Pbcn (60)	6.393(1)	4.362(1)	4.543(1)	90	90	90
No structure type (Al-O) Class_Num_10	23660	$(Al_2O_3)_{5.333}$	$P6_3mc$ (186)	5.544(1)	5.544(1)	9.024(1)	90	90	120
No structure type (Al-O) Class_Num_11	40200	Al $Al_{1.67}O_4$	P-4m2 (115)	5.599(10)	5.599(10)	23.657(50)	90	90	90
Fluorite-CaF_2, Li_2O (Li-O) Class_Num_12	173180	Li_2O	Fm-3m (225)	4.614(1)	4.614(1)	4.614(1)	90	90	90
	173193	Li_2O	Fm-3m (225)	4.6124(1)	4.6124(1)	4.6124(1)	90	90	90
	173206	Li_2O	Fm-3m (225)	4.6128(4)	4.6128(4)	4.6128(4)	90	90	90
	182024	Li_2O	Fm-3m (225)	4.728(5)	4.728(5)	4.728(5)	90	90	90
	182025	Li_2O	Fm-3m (225)	4.764(5)	4.764(5)	4.764(5)	90	90	90
	182026	Li_2O	Fm-3m (225)	4.782(5)	4.782(5)	4.782(5)	90	90	90
	182027	Li_2O	Fm-3m (225)	4.807(5)	4.807(5)	4.807(5)	90	90	90
	182028	Li_2O	Fm-3m (225)	4.837(5)	4.837(5)	4.837(5)	90	90	90
	22402	Li_2O	Fm-3m (225)	4.61	4.61	4.61	90	90	90
	257372	Li_2O	Fm-3m (225)	4.61549(5)	4.61549(5)	4.61549(5)	90	90	90
	54368	Li_2O	Fm-3m (225)	4.610(5)	4.610(5)	4.610(5)	90	90	90
	57411	Li_2O	Fm-3m (225)	4.623	4.623	4.623	90	90	90
	60431	Li_2O	Fm-3m (225)	4.628	4.628	4.628	90	90	90
	642216	Li_2O	Fm-3m (225)	4.693(5)	4.693(5)	4.693(5)	90	90	90
Li_2O_2 (Li-O) Class_Num_13	25530	Li_2O_2	$P6_3/mmc$ (194)	3.142	3.142	7.65	90	90	120
No structure type (Li-O) Class_Num_14	24143	Li_2O_2	P-6 (174)	6.305	6.305	7.71	90	90	120
No structure type (Li-O) Class_Num_15	108886	Li_2O	R-3mH (166)	3.624	3.624	7.97	90	90	120
	105548	SrO	Fm-3m (225)	5.160(2)	5.16	5.16	90	90	90

NaCl (Sr-O) Class_Num_16	109461	SrO	Fm-3m (225)	5.1615(3)	5.1615(3)	5.1615(3)	90	90	90
	163625	SrO	Fm-3m (225)	5.16132(13)	5.16132(13)	5.16132(13)	90	90	90
	249178	SrO	Fm-3m (225)	5.1326	5.1326	5.1326	90	90	90
	26960	SrO	Fm-3m (225)	5.124(10)	5.124	5.124	90	90	90
	28904	SrO	Fm-3m (225)	5.1396	5.1396	5.1396	90	90	90
	52276	SrO	Fm-3m (225)	5.154(2)	5.154	5.154	90	90	90
CaC_2 (Sr-O) Class_Num_17	24249	SrO_2	I4/mmm (139)	3.55	3.55	6.55	90	90	90
	647474	SrO_2	I4/mmm (139)	3.557	3.557	6.56	90	90	90
	74967	$SrO_{1.95}$	I4/mmm (139)	3.5626(3)	3.5626(3)	6.6159(6)	90	90	90
	88749	$SrO_{1.978}$	I4/mmm (139)	3.5630(2)	3.5630(2)	6.616(1)	90	90	90
	88750	$SrO_{1.962}$	I4/mmm (139)	3.5619(3)	3.5619(3)	6.576(1)	90	90	90
	88751	$SrO_{1.9}$	I4/mmm (139)	3.5585(4)	3.5585(4)	6.563(1)	90	90	90
$LiAlO_2$-gamma (Li-Al-O) Class_Num_18	23815	$LiAlO_2$	$P4_12_12$ (92)	5.1687(5)	5.1687(5)	6.2679(6)	90	90	90
	30249	$AlLiO_2$	$P4_12_12$ (92)	5.17	5.17	6.295	90	90	90
	430184	$LiAlO_2$	$P4_12_12$ (92)	5.1685(4)	5.1685(4)	6.2565(8)	90	90	90
	430185	$LiAlO_2$	$P4_12_12$ (92)	5.250(10)	5.250(10)	6.340(10)	90	90	90
	430357	$LiAlO_2$	$P4_12_12$ (92)	5.15885(17)	5.15885(17)	6.2700(3)	90	90	90
	430358	$LiAlO_2$	$P4_12_12$ (92)	5.1965(2)	5.1965(2)	6.3464(3)	90	90	90
	430359	$LiAlO_2$	$P4_12_12$ (92)	5.2252(2)	5.2252(2)	6.4038(3)	90	90	90
Li_5AlO_4 (Li-Al-O) Class_Num_19	1037	Li_5AlO_4	PmmnZ (59)	6.42	6.302	4.62	90	90	90
	16229	Li_5AlO_4	PmmnZ (59)	6.424(3)	6.305(3)	4.623(2)	90	90	90
spinel-$LiFe_5O_8$ (Li-Al-O) Class_Num_20	10480	$LiAl_5O_8$	$P4_332$ (212)	7.908(2)	7.908(2)	7.908(2)	90	90	90
	83016	$LiAl_5O_8$	$P4_332$ (212)	7.903(5)	7.903(5)	7.903(5)	90	90	90
Spinel-defect (Li-Al-O) Class_Num_21	83017	$(Li_{0.25}Al_{0.75})_2(Li_{0.75}Al_{1.25})_2O_7$	Fd-3mS (227)	7.910(5)	7.910(5)	7.910(5)	90	90	90
Delafossite-$NaCrS_2$ (Li-Al-O) Class_Num_22	28288	$LiAlO_2$	R-3mH (166)	2.8003(6)	2.8003(6)	14.216(3)	90	90	120
$LiFeO_2$-alpha (Li-Al-O) Class_Num_23	99517	$LiAlO_2$	$I4_1$/amdZ (141)	3.8866(8)	3.8866(8)	8.3001(13)	90	90	90
Li_5GaO_4-alpha (Li-Al-O) Class_Num_24	42697	Li_5AlO_4	Pbca (61)	9.087(3)	8.947(3)	9.120(3)	90	90	90
$SrAl_2O_4$ (Sr-Al-O) Class_Num_25	160296	$Sr(Al_2O_4)$	$P12_11$ (4)	8.44365(9)	8.82245(8)	5.15964(6)	90	93.411(1)	90
	160297	$Sr(Al_2O_4)$	$P12_11$ (4)	8.5066(5)	8.8898(5)	5.1732(3)	90	92.875(4)	90

	26466	$SrAl_2O_4$	$P12_11$ (4)	8.447	8.816	5.163	90	93.42	90
	291357	$SrAl_2O_4$	$P12_11$ (4)	8.43904(1)	8.81425(2)	5.156355(9)	90	93.4094(1)	90
	291361	$SrAl_2O_4$	$P12_11$ (4)	8.41634(8)	8.78882(8)	5.1565(5)	90	93.5079(9)	90
M-type-ferrite#$CaAl_{12}O_{19}$ (Sr-Al-O) Class_Num_26	184967	$Sr(Al_{12}O_{19})$	$P6_3/mmc$ (194)	5.564	5.564	22.002	90	90	120
	2006	$SrO(Al_2O_3)_6$	$P6_3/mmc$ (194)	5.562(2)	5.562(2)	21.9719(50)	90	90	120
	239757	$Al_{12}SrO_{19}$	$P6_3/mmc$ (194)	5.57661	5.57661	22.143(1)	90	90	120
	69020	$SrAl_{12}O_{19}$	$P6_3/mmc$ (194)	5.5666(2)	5.5666(2)	22.0018(8)	90	90	120
$Sr_4Al_{14}O_{25}$ (Sr-Al-O) Class_Num_27	258392	$Sr_4Al_{14}O_{25}$	Pmma (51)	24.7703(84)	8.4797(31)	4.8833(17)	90	90	90
	27744	$Sr_4Al_4O_2(Al_{10}O_{23})$	Pmma (51)	24.785(1)	8.487(2)	4.886(1)	90	90	90
	88527	$(SrO)_4(Al_2O_3)_7$	Pmma (51)	24.74509(20)	8.4735(6)	4.8808(1)	90	90	90
$BaMgSiO_4$ (Sr-Al-O) Class_Num_28	153164	$Sr(Al_2O_4)$	$P6_3$ (173)	8.9260(3)	8.9260(3)	8.4985(2)	90	90	120
	160298	$Sr(Al_2O_4)$	$P6_3$ (173)	8.9291(1)	8.9291(1)	8.4963(4)	90	90	120
	160299	$Sr(Al_2O_4)$	$P6_3$ (173)	8.9349(1)	8.9349(1)	8.5109(3)	90	90	120
$BaAl_2O_4$ (Sr-Al-O) Class_Num_29	160300	$Sr(Al_2O_4)$	$P6_322$ (182)	5.1666(1)	5.1666(1)	8.5485(3)	90	90	120
	160301	$Sr(Al_2O_5)$	$P6_322$ (182)	5.1765(1)	5.1765(1)	8.5758(1)	90	90	120
$Ca_9Al_6O_{18}$ (Sr-Al-O) Class_Num_30	66062	$Sr_9(Al_6O_{18})$	Pa-3 (205)	15.8476(2)	15.8476(2)	15.8476(2)	90	90	90
	71860	$Sr_3Al_2O_6$	Pa-3 (205)	15.8556(4)	15.8556(4)	15.8556(4)	90	90	90
$CaAl_4O_7$ (Sr-Al-O) Class_Num_31	16751	$(SrO)(Al_2O_3)_2$	C12/c1 (15)	13.04	9.01	5.55	90	106.502	90
	2817	$SrAl_4O_7$	C12/c1 (15)	13.0389(90)	9.0113(45)	5.5358(27)	90	106.7	90
No structure type (Sr-Al-O) Class_Num_32	200671	$Sr_7Al_{12}O_{25}$	P3 (143)	17.91	17.91	7.16	90	90	120
	57177	$Sr_7Al_{12}O_{25}$	P-3 (147)	17.91	17.91	7.16	90	90	120
$CaAlB_3O_7$ (Sr-Al-O) Class_Num_33	34803	$Sr(Al_4O_7)$	Cmma (67)	8.085(5)	11.845(8)	4.407(3)	90	90	90
$Sr_{0.5}Al_{11}O_{17}$ (Sr-Al-O) Class_Num_34	108851	$Sr_{0.5}Al_{11}O_{17}$	R-3mH (166)	5.622	5.622	33.5	90	90	120
$Sr_{10}Ga_6O_{19}$ (Sr-Al-O) Class_Num_35	95535	$Sr_{10}Al_6O_{19}$	C12/c1 (15)	34.5823(21)	7.8460(6)	15.7485(9)	90	103.68(1)	90
No structure type (Sr-Al-O) Class_Num_36	97713	$Sr_2(Al_6O_{11})$	Pnnm (58)	21.9145(8)	4.8843(2)	8.4039(3)	90	90	90
No structure type (Sr-Li-Al-O) Class_Num_37	-	Sr_2LiAlO_4	$P12_1/m1$ (11)	5.81998	5.63362	6.65907	90	106.483	90

Supplementary Table 4 A comparison between the height fraction and the weight fraction, which was correctly measured using real experimental XRD pattern data for $SrAl_2O_4$-SrO-Al_2O_3 and Li_2O-SrO-Al_2O_3 mixture systems.

Compositional space	Sample No.	Weight fraction			Mol. fraction			Height fraction		
		Li_2O	SrO	Al_2O_3	Li_2O	SrO	Al_2O_3	Li_2O	SrO	Al_2O_3
Li_2O-SrO-Al_2O_3	0	17.80%	12.30%	69.90%	42.55%	8.48%	48.97%	13.76%	27.72%	58.52%
	1	31.72%	23.98%	44.29%	61.46%	13.40%	25.15%	39.73%	44.01%	16.27%
	2	20.61%	38.55%	40.84%	47.17%	25.44%	27.39%	29.46%	59.49%	11.05%
	3	12.07%	13.32%	74.62%	31.95%	10.17%	57.88%	26.93%	30.42%	42.64%
	4	23.62%	31.10%	45.28%	51.51%	19.56%	28.94%	30.24%	54.52%	15.24%
	5	12.68%	30.44%	56.88%	33.26%	23.02%	43.72%	10.57%	63.47%	25.96%
	6	25.82%	29.23%	44.95%	54.45%	17.77%	27.78%	25.93%	56.73%	17.34%
	7	12.50%	17.47%	70.02%	32.85%	13.24%	53.92%	14.72%	46.77%	38.51%
	8	13.50%	8.17%	78.33%	34.78%	6.07%	59.15%	16.69%	31.71%	51.60%
	9	16.68%	9.06%	74.26%	40.63%	6.36%	53.01%	19.71%	31.34%	48.95%
	10	32.03%	5.33%	62.64%	61.69%	2.96%	35.35%	48.19%	16.85%	34.96%
	11	9.99%	8.91%	81.10%	27.50%	7.07%	65.43%	12.97%	29.43%	57.59%
	12	7.67%	20.02%	72.32%	22.14%	16.67%	61.19%	16.97%	49.05%	33.98%
	13	13.11%	21.87%	65.02%	34.08%	16.39%	49.53%	13.22%	52.65%	34.13%
	14	23.02%	25.16%	51.82%	50.64%	15.96%	33.40%	23.23%	53.23%	23.54%
	15	21.80%	24.82%	53.38%	48.88%	16.05%	35.07%	24.80%	51.67%	23.53%
	16	15.44%	23.40%	61.17%	38.49%	16.82%	44.69%	11.94%	59.16%	28.89%
	17	22.22%	16.40%	61.39%	49.44%	10.52%	40.03%	9.69%	59.11%	31.20%
	18	14.53%	22.87%	62.60%	36.81%	16.71%	46.48%	22.40%	32.37%	45.23%
	19	15.87%	23.45%	60.68%	39.27%	16.73%	44.00%	22.13%	54.68%	23.19%
	20	21.98%	11.51%	66.50%	49.08%	7.41%	43.51%	16.21%	50.42%	33.38%
	21	22.64%	17.82%	59.54%	50.06%	11.36%	38.58%	24.20%	31.14%	44.66%
	22	25.45%	8.19%	66.35%	53.86%	5.00%	41.15%	17.15%	44.78%	38.07%
	23	39.25%	32.13%	28.63%	68.97%	16.28%	14.74%	21.07%	66.55%	12.38%
	24	13.74%	14.97%	71.29%	35.28%	11.08%	53.64%	14.95%	44.61%	40.44%
	25	29.67%	28.52%	41.82%	59.16%	16.40%	24.44%	25.36%	57.52%	17.11%
	26	19.02%	9.44%	71.54%	44.54%	6.37%	49.09%	29.83%	27.67%	42.50%
	27	37.53%	19.07%	43.40%	67.32%	9.86%	22.81%	38.11%	39.02%	22.87%
	28	9.91%	3.95%	86.14%	27.31%	3.14%	69.56%	29.86%	13.96%	56.18%
	29	14.58%	5.46%	79.96%	36.83%	3.98%	59.19%	25.23%	16.98%	57.79%
	30	14.88%	2.90%	82.22%	37.38%	2.10%	60.52%	25.68%	12.69%	61.63%

	31	9.07%	16.88%	74.04%	25.45%	13.66%	60.89%	9.92%	48.55%	41.53%
	32	19.15%	3.55%	77.30%	44.71%	2.39%	52.89%	21.58%	15.52%	62.89%
	33	28.14%	21.71%	50.15%	57.32%	12.75%	29.93%	39.69%	39.82%	20.49%
	34	17.02%	5.31%	77.67%	41.20%	3.71%	55.10%	27.60%	16.42%	55.98%
	35	14.79%	14.83%	70.39%	37.26%	10.77%	51.97%	15.57%	42.77%	41.66%
	36	16.94%	4.78%	78.28%	41.06%	3.34%	55.60%	29.32%	15.92%	54.76%
	37	32.69%	40.49%	26.82%	62.59%	22.36%	15.05%	29.90%	60.87%	9.24%
	38	22.18%	33.31%	44.51%	49.48%	21.43%	29.10%	15.34%	66.18%	18.48%
	39	34.99%	18.18%	46.83%	64.85%	9.72%	25.44%	30.50%	45.90%	23.60%
	40	13.35%	16.35%	70.29%	34.53%	12.19%	53.28%	10.70%	45.91%	43.39%
	41	28.36%	7.55%	64.09%	57.50%	4.41%	38.08%	45.15%	20.00%	34.85%
	42	18.84%	9.64%	71.52%	44.25%	6.53%	49.22%	23.30%	28.67%	48.03%
	43	13.74%	28.89%	57.38%	35.33%	21.42%	43.24%	9.05%	64.75%	26.20%
	44	15.27%	8.36%	76.37%	38.12%	6.02%	55.87%	18.22%	27.92%	53.86%
	45	15.81%	16.91%	67.28%	39.13%	12.07%	48.80%	31.32%	26.64%	42.04%
	46	14.45%	27.68%	57.87%	36.68%	20.26%	43.05%	18.22%	56.83%	24.95%
	47	18.47%	16.38%	65.16%	43.68%	11.17%	45.15%	21.44%	42.33%	36.23%
	48	34.11%	27.37%	38.53%	64.00%	14.81%	21.19%	31.19%	52.67%	16.15%
	49	6.32%	15.86%	77.83%	18.75%	13.57%	67.68%	8.77%	46.12%	45.11%

Compositional space	Sample No.	Weight fraction			Mol. fraction			Height fraction		
		$SrAl_2O_4$	SrO	Al_2O_3	$SrAl_2O_4$	SrO	Al_2O_3	$SrAl_2O_4$	SrO	Al_2O_3
$SrAl_2O_4$-SrO-Al_2O_3	0	62.02%	6.09%	31.88%	44.82%	8.73%	46.45%	32.04%	28.17%	39.79%
	1	33.74%	6.76%	59.49%	20.19%	8.03%	71.78%	22.60%	23.28%	54.11%
	2	61.79%	10.88%	27.33%	44.62%	15.59%	39.79%	26.74%	37.90%	35.35%
	3	61.18%	11.52%	27.30%	43.99%	16.43%	39.58%	26.21%	38.68%	35.11%
	4	73.45%	2.63%	23.92%	57.88%	4.11%	38.01%	20.96%	55.57%	23.46%
	5	68.41%	10.78%	20.82%	51.91%	16.23%	31.86%	30.01%	36.13%	33.86%
	6	56.04%	16.67%	27.29%	38.88%	22.95%	38.17%	23.92%	45.14%	30.94%
	7	21.29%	12.52%	66.19%	11.85%	13.83%	74.31%	11.55%	39.89%	48.56%
	8	22.73%	12.79%	64.48%	12.76%	14.25%	72.99%	12.77%	37.93%	49.30%
	9	57.98%	7.89%	34.13%	40.70%	10.99%	48.31%	31.21%	28.20%	40.59%
	10	85.77%	3.93%	10.30%	75.02%	6.82%	18.16%	40.51%	23.54%	35.94%
	11	31.92%	6.78%	61.29%	18.89%	7.96%	73.14%	19.98%	24.44%	55.59%
	12	56.51%	12.44%	31.05%	39.30%	17.16%	43.54%	27.54%	38.40%	34.06%
	13	58.33%	13.17%	28.50%	41.10%	18.41%	40.49%	24.61%	41.12%	34.27%
	14	81.75%	8.68%	9.57%	69.12%	14.56%	16.32%	35.92%	32.64%	31.43%

	15	82.84%	5.52%	11.64%	70.65%	9.34%	20.01%	37.98%	26.38%	35.64%
	16	68.13%	8.52%	23.35%	51.57%	12.79%	35.64%	28.50%	38.56%	32.94%
	17	55.45%	9.48%	35.07%	38.25%	12.97%	48.78%	27.87%	32.77%	39.37%
	18	39.18%	14.78%	46.04%	24.29%	18.18%	57.54%	18.04%	45.23%	36.73%
	19	22.47%	18.96%	58.57%	12.61%	21.11%	66.28%	10.45%	48.01%	41.54%
	20	38.35%	14.62%	47.03%	23.65%	17.88%	58.47%	18.19%	42.14%	39.68%
	21	39.08%	40.70%	20.22%	24.33%	50.28%	25.39%	12.20%	71.71%	16.08%
	22	58.08%	1.95%	39.96%	40.75%	2.71%	56.53%	31.21%	20.66%	48.12%
	23	71.86%	12.02%	16.12%	56.05%	18.60%	25.35%	29.86%	38.48%	31.67%
	24	59.91%	5.00%	35.08%	42.62%	7.06%	50.32%	29.27%	28.67%	42.06%
	25	79.23%	8.91%	11.86%	65.58%	14.63%	19.79%	34.90%	33.58%	31.51%
	26	47.90%	14.82%	37.27%	31.42%	19.29%	49.29%	22.74%	40.81%	36.45%
	27	61.07%	9.28%	29.64%	43.86%	13.22%	42.92%	30.61%	32.75%	36.64%
	28	63.14%	3.06%	33.80%	45.97%	4.42%	49.61%	31.83%	23.64%	44.54%
	29	62.37%	16.42%	21.20%	45.30%	23.66%	31.04%	24.31%	48.27%	27.41%
	30	48.76%	13.78%	37.46%	32.16%	18.03%	49.81%	20.87%	43.42%	35.71%
	31	77.46%	2.86%	19.68%	63.07%	4.62%	32.31%	41.68%	20.15%	38.18%
	32	43.59%	23.29%	33.12%	27.84%	29.51%	42.65%	17.97%	52.90%	29.13%
	33	65.46%	10.00%	24.53%	48.58%	14.72%	36.70%	28.04%	39.84%	32.12%
	34	51.83%	30.87%	17.29%	35.04%	41.40%	23.57%	15.03%	65.60%	19.37%
	35	39.85%	8.41%	51.74%	24.77%	10.37%	64.85%	21.11%	31.90%	46.99%
	36	54.55%	12.30%	33.15%	37.42%	16.74%	45.85%	26.75%	36.81%	36.44%
	37	36.16%	43.58%	20.26%	22.12%	52.89%	24.99%	9.94%	75.68%	14.38%
	38	69.54%	4.60%	25.86%	53.16%	6.98%	39.86%	35.70%	22.08%	42.22%
	39	54.12%	14.49%	31.39%	37.03%	19.67%	43.30%	23.94%	44.02%	32.04%
	40	45.94%	14.73%	39.34%	29.74%	18.92%	51.35%	20.20%	42.83%	36.97%
	41	30.55%	16.30%	53.15%	17.96%	19.02%	63.02%	14.73%	47.40%	37.87%
	42	64.78%	7.95%	27.27%	47.80%	11.64%	40.57%	30.48%	32.09%	37.43%
	43	67.49%	8.79%	23.72%	50.84%	13.14%	36.03%	31.43%	31.54%	37.03%
	44	70.57%	5.65%	23.77%	54.41%	8.64%	36.95%	34.56%	26.79%	38.66%
	45	55.34%	7.00%	37.66%	38.12%	9.57%	52.31%	28.37%	28.74%	42.89%
	46	33.75%	18.17%	48.08%	20.24%	21.62%	58.14%	14.93%	49.13%	35.95%
	47	84.15%	10.62%	5.23%	72.69%	18.20%	9.11%	36.91%	33.72%	29.37%
	48	74.99%	12.62%	12.39%	59.99%	20.03%	19.98%	31.22%	39.25%	29.53%
	49	57.08%	28.77%	14.15%	40.00%	40.00%	19.99%	30.81%	42.26%	26.92%
