# Supplementary material for: A deep-learning technique for phase identification in multiphase inorganic compounds using synthetic XRD powder patterns
Source: Nat Commun. 2020 Jan 3;11:86. doi: 10.1038/s41467-019-13749-3 (PMC6941984; doi:10.1038/s41467-019-13749-3)
Supplement: Supplementary file 2 — Description of Additional Supplementary Files [file 41467_2019_13749_MOESM2_ESM.pdf]

Description of Additional Supplementary Files

File name: Supplementary Data 1

Description: Weight dataset 1. Fully trained weights and biases for Simple phase identification code

File name: Supplementary Data 2

Description: Weight dataset 2. Fully trained weights and biases for 3_Level_Fraction_Prediction code
